# Solvatomorphism in a series of copper(II) complexes with the 5-phenyl­imidazole/perchlorate system as ligands

**DOI:** 10.1107/S2052520624005948

**Published:** 2024-07-30

**Authors:** Edward Loukopoulos, Constantina Papatriantafyllopoulou, Eleni Moushi, Alexandros A. Kitos, Anastasios J. Tasiopoulos, Spyros P. Perlepes, Vassilios Nastopoulos

**Affiliations:** a Department of Chemistry University of Patras Patras 26504 Greece; b Department of Chemistry University of Cyprus Nicosia 1678 Cyprus; CSIR–National Chemical Laboratory, India

**Keywords:** solvatomorphism/pseudopolymorphism, crystal engineering, phenyl­imidazoles, X-ray structure determination, metallosupramolecular chemistry, C-substituted imidazoles, X-ray diffraction

## Abstract

In the course of an investigation of the supramolecular behaviour of copper(II) complexes with the 5-phenyl­imidazole/perchlorate ligand system (‘blend’) remarkable solvatomorphism has been observed. By employing a variety of crystallization solvents (polar protic, polar/non-polar aprotic), a series of 12 crystalline solvatomorphs with the general formula [Cu(ClO_4_)_2_(*L*H)_4_]·*x*(solvent) have been obtained [*L*H = 5-phenyl­imidazole, *x*(solvent) = 3.3(H_2_O) (**1**), 2(methanol) (**2**), 2(ethanol) (**3**), 2(1-propanol) (**4**), 2(2-propanol) (**5**), 2(2-butanol) (**6**), 2(di­methyl­formamide) (**7**), 2(acetone) (**8**), 2(tetra­hydro­furane) (**9**), 2(1,4-dioxane) (**10**), 2(ethyl acetate) (**11**) and 1(di­ethyl ether) (**12**)]. The structures have been solved using single-crystal X-ray diffraction and the complexes were characterized by thermal analysis and infrared spectroscopy. The solvatomorphs are isostructural (triclinic, *P*1), with the exception of compound **9** (monoclinic, *P*2_1_/*n*). The supramolecular structures and the role of the various solvents is discussed. All potential hydrogen-bond functionalities, both of the [Cu(ClO_4_)_2_(*L*H)_4_] units and of the solvents, are utilized in the course of the crystallization process. The supramolecular assembly in all structures is directed by strong recurring N_imidazole_–H⋯O_perchlorate_ motifs leading to robust scaffolds composed of the [Cu(ClO_4_)_2_(*L*H)_4_] host complexes. The solvents are located in channels and, with the exception of the disordered waters in **1** and the di­ethyl ether in **12**, participate in hydrogen-bonding formation with the [Cu(ClO_4_)_2_(*L*H)_4_] complexes, serving as both hydrogen-bond acceptors and donors (for the polar protic solvents in **2**–**6**), or solely as hydrogen-bond acceptors (for the polar/non-polar aprotic solvents in **7**–**11**), linking the complexes and contributing to the stability of the crystalline compounds.

## Abstract 2

The reactions of Cu(ClO_4_)_2_·6H_2_O and *L*′H in a variety of solvents have provided access to 12 solvatomorphs of the general formula [Cu(ClO_4_)_2_(*L*H)_4_]·*x*(solvent); the effect of the solvents on the packing of the complexes is critically discussed.

## 1. Introduction

The term solvatomorphism (often called pseudopolymorphism) is used for designating solvate diversity of a particular host compound (Ibragimov, 2007 ▸), or in other words, refers to the ability of a compound to yield crystal structures with unit cells that differ in their elemental composition through the inclusion of various amounts or types of solvent molecules (Brittain, 2012 ▸). In the past, a number of scientific arguments have been put forward by experts in the field regarding the use of the terms solvatomorph and pseudopolymorph (also including terms such as supramolecular isomer, false polymorph or quasipolymorph) that could describe the above category of crystalline structures (Threlfall, 1995 ▸; Nangia & Desiraju, 1999 ▸; Seddon, 2004 ▸; Desiraju, 2004 ▸; Bernstein, 2005 ▸, 2011 ▸; Nangia, 2006 ▸; Brittain, 2012 ▸; Stöger *et al.*, 2012 ▸; du Plessis & Barbour, 2012 ▸). It seems that the term pseudopolymorphism is more widely used in the literature; however, in the present study the term solvatomorphism is used to emphasize that the crystal structures differ in the type of solvent used in their synthesis reactions. Polymorphism (Sarma & Desiraju, 1999 ▸) and solvatomorphism are interesting topics in crystal engineering in relation to the implications on the crystal packing and the potential impact on the properties (chemical, physical *etc*.) of crystalline solids (Nangia & Desiraju, 2019 ▸; Ranjan *et al.*, 2020 ▸). With respect to solvatomorphism, the effect of the solvent on the organization of supramolecular systems (by forming synthons based on solute–solvent specific interactions) is of great interest, both for academic studies and practical applications (Görbitz & Hersleth, 2000 ▸; Hao *et al.*, 2005 ▸; Mondal & Howard, 2005 ▸). For example, for some active pharmaceutical ingredients (APIs) solvatomorphs could be the ultimate pharmaceutical form for clinical use because of their advantages in improving the solubility or stability of APIs and, therefore, the performance of the drug (Yuan *et al.*, 2020 ▸); in biological systems where a modification of the protein solvent environment by denaturation results in changes in their biological activities; in the self-assembly of MOFs (Köppen *et al.*, 2018 ▸; Seetharaj *et al.*, 2019 ▸); in organic and inorganic functional nanomaterials (Yamada *et al.*, 2006 ▸; Bernard *et al.*, 2018 ▸) *etc*. However, solvate/hydrate formation is a rather unpredictable process, as we are not able to tell in advance whether a solvent or a water molecule will crystallize with the solute molecule (Juarez-Garrido *et al.*, 2022 ▸; Glasser, 2019 ▸; Li & Du, 2011 ▸).

**Figure 1:**
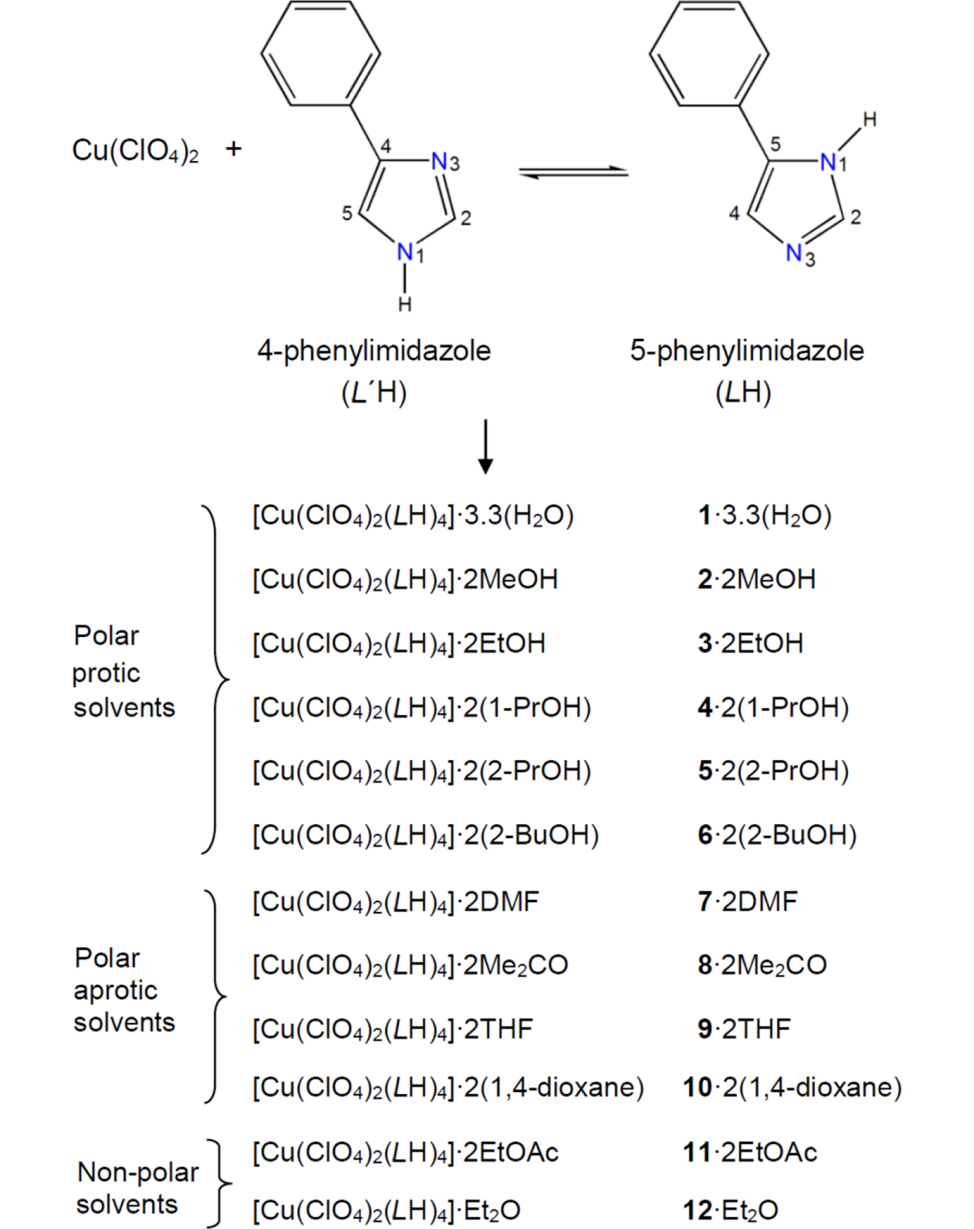
The 4-phenyl­imidazole (*L*′H) ligand and the crystallographically determined formulae of the isolated solvatomorphs with the general composition [Cu(ClO_4_)_2_(*L*H)_4_]·*x*(solvent). The initially used 4-phenyl­imidazole ligand is present in all complexes as its 5-phenyl­imidazole tautomeric form (*L*H). MeOH = methanol, EtOH = ethanol, 1-PrOH = 1-propanol, 2-PrOH = 2-propanol, 2-BuOH = 2-butanol, DMF = dimethylformamide, Me_2_CO = acetone, THF = tetrahydrofuran, EtOAc = ethyl acetate, Et_2_O = diethyl ether .

In recent years we have studied a series of 3*d* metal complexes with substituted imidazole ligands to investigate the patterns followed in the assembly process of these materials, with particular interest in the intermolecular – both strong and weak – interactions (Kounavi *et al.*, 2009 ▸, 2012*a* ▸,*b* ▸, 2015 ▸; Kitos *et al.*, 2016 ▸; Duros *et al.*, 2019 ▸). With respect to this, we sought in the present study to use the relatively small and versatile 4-phenyl­imidazole ligand (*L*′H) in combination with the perchlorate salt of copper(II). Copper(II) is a special metal ion in coordination chemistry with a flexible coordination sphere. It has been proven popular in metallo­supra­molecular chemistry as it favours a variety of coordination numbers and geometries (Cotton *et al.*, 1999 ▸; Reinen, 1983 ▸) that can be realized by properly selected ligands and reaction conditions. Due to its 3*d*^9^ configuration, square planar or distorted tetrahedral (four-coordinate), square pyramidal or trigonal bipyramidal (five-coordinate), as well as distorted octahedral (six-coordinate) geometries characterize the coordination chemistry of copper(II). Imidazole and its derivatives have played an important role in coordination chemistry (Constable, 1996 ▸; Chen, 2016 ▸). They are also interesting ligands in bioinorganic (Lippard & Berg, 1994 ▸; Roat-Malone, 2002 ▸) and metallosupramolecular chemistry, employed, for example, in the synthesis of clusters, coordination polymers and MOFs (Desiraju, 1995 ▸; Steed & Atwood, 2009 ▸). The *L*′H ligand coordinates *via* its pyridine-type N3 atom towards mononuclear species (Fig. 1 ▸), has a hydrogen-bond donor (the pyrrolic-type N1 atom) enabling the formation of strong supramolecular motifs (Schmidt, 1971 ▸; Desiraju, 1995 ▸; Steiner, 2002 ▸; Aakeröy *et al.*, 2010 ▸; Dunitz & Gavezzotti, 2012 ▸) and is also capable of forming π⋯π stackings (Hunter & Sanders, 1990 ▸; Janiak, 2000 ▸; Nishio *et al.*, 2012 ▸) through its five- and six-membered aromatic rings. The perchlorate ion has been considered as a rather weak ligand to the transition metal ions (Sekizaki, 1981 ▸). However, it sometimes acts as a unidentate ligand (thus excluding the possibility of forming coordination polymers) in the presence of organic ligands. It seemed therefore interesting to study its coordination ability with regard to the present synthetic system, as well as its spatial role in the motif formation and packing organization of the complexes produced.

Remarkable solvatomorphism was obtained during crystallization experiments for the above general reaction system. Utilizing a variety of crystallization solvents (polar protic, polar/non-polar aprotic), a series of 12 crystalline solvato­morphs with the general formula [Cu(ClO_4_)_2_(*L*H)_4_]·*x*(solvent) (Fig. 1 ▸) have been isolated and structurally characterized. Their molecular and supramolecular structures have been studied and compared to understand the effect of the solvent molecules properties, such as size, shape and intermolecular interactions, in the solvatomorph formation.

## 2. Experimental

All reagents and starting materials were reagent grade, purchased from Aldrich and used as received. All manipulations were performed under aerobic conditions. Microanalyses (C, H, N) were performed by the University of Patras microanalytical service. FT-IR spectra (4000–400 cm^−1^) were recorded using a Perkin–Elmer PC 16FT-IR spectrometer with samples prepared as KBr pellets; the spectra were recorded on analytically pure, well dried samples, and not on the as-isolated crystals. Thermogravimetric analysis (TGA) studies were performed with a Shimadzu TGA 50 thermogravimetric analyser at a 10°C min^−1^ increase rate under air.

*Safety note*: Perchlorate salts are potentially explosive; such compounds should be synthesized and used in small quantities, and treated with utmost care at all times.

### 2.1. Preparation of complexes

#### 2.1.1. [Cu(ClO_4_)_2_(*L*H)_4_]·3.3(H_2_O) [1·3.3(H_2_O)]

*L′*H (0.173 g, 1.2 mmol) and Cu(ClO_4_)_2_·6H_2_O (0.111 g, 0.3 mmol) were dissolved in a mixture of CH_2_Cl_2_ (25 ml) and MeCN (5 ml) and stirred for 30 min. The resultant turquoise solution was filtered and then layered with 50 ml of *n*-hexane/Et_2_O (1:2 *v*/*v* ratio) to produce purple prismatic crystals after two days. The crystals were collected by filtration and dried in a vacuum desiccator over silica gel. The overall yield was 35% (based on Cu^II^). Analysis calculated for C_36_H_38.6_Cl_2_CuN_8_O_11.3_ [**1**·3.3(H_2_O)] (found values in parentheses): C 48.01 (48.35), H 4.35 (4.22), N 12.45 (12.30)%. Selected IR bands (KBr, cm^−1^): 3430*s*, 3320*m*, 3140*s*, 3018*w*, 1637*w*, 1612*w*, 1592*m*, 1486*m*, 1450*m*, 1427*w*, 1350*w*, 1269*w*, 1245*w*, 1119*s*, 1088*s*, 965*m*, 956*m*, 840*m*, 758*s*, 690*s*, 644*m*, 622*s*, 514*w*, 450*w*.

#### 2.1.2. [Cu(ClO_4_)_2_(*L*H)_4_]·2MeOH (2·2MeOH)

A solution of *L*′H (0.173 g, 1.2 mmol) and Cu(ClO_4_)_2_·6H_2_O (0.111 g, 0.3 mmol) in MeOH (25 ml) was stirred for 30 min. The resultant turquoise solution was filtered and then layered with 50 ml of Et_2_O (1:2 *v*/*v* ratio) to produce red prismatic crystals after two days in a 65% yield (based on Cu^II^). The crystals were collected by filtration and dried in a vacuum desiccator over silica gel. Analysis calculated for C_36_H_32_Cl_2_CuN_8_O_8_ (**2**) (found values in parentheses): C 51.53 (51.77), H 3.84 (3.72), N 13.35 (13.50)%. Selected IR bands (KBr, cm^−1^): 3300*m*, 3144*m*, 3022*w*, 1654*w*, 1636*w*, 1614*w*, 1590*m*, 1488*m*, 1452*m*, 1424*w*, 1356*w*, 1274*w*, 1246*w*, 1122*s*, 1090*s*, 966*m*, 952*m*, 838*m*, 760*s*, 692*s*, 648*m*, 624*s*, 512*w*, 452*w*.

#### 2.1.3. [Cu(ClO_4_)_2_(*L*H)_4_]·2EtOH (3·2EtOH)

A solution of *L*′H (0.180 g, 1.25 mmol) and Cu(ClO_4_)_2_·6H_2_O (0.185 g, 0.5 mmol) in EtOH (25 ml) was stirred for 30 min. The resultant turquoise solution was filtered, then layered with 50 ml of *n*-hexane (1:2 *v*/*v* ratio) to produce purple prismatic crystals after two days in a 55% yield (based on *L*H). The crystals were collected by filtration and dried in a vacuum desiccator over silica gel. Analysis calculated for C_36_H_32_Cl_2_CuN_8_O_8_ (**3**) (found values in parentheses): C 51.53 (51.82), H 3.84 (3.71), N 13.35 (13.58)%. Selected IR bands (KBr, cm^−1^): 3420*mb*, 3314*m*, 3142*s*, 3022*w*, 1612*w*, 1590*w*, 1490*m*, 1456*w*, 1424*w*, 1358*w*, 1274*w*, 1234*w*, 1122*s*, 1088*s*, 966*m*, 952*m*, 840*m*, 762*s*, 692*m*, 648*m*, 626*s*, 508*w*, 452*w*.

#### 2.1.4. [Cu(ClO_4_)_2_(*L*H)_4_]·2(1-PrOH) [4·2(1-PrOH)]

A solution of *L*′H (0.180 g, 1.25 mmol) and Cu(ClO_4_)_2_·6H_2_O (0.185 g, 0.5 mmol) in 1-PrOH (25 ml) was stirred for 30 min. The resultant turquoise solution was filtered. Upon slow evaporation of the filtrate, purple prismatic crystals were obtained after four days, collected by filtration and dried in a vacuum desiccator over anhydrous CaCl_2_; the yield was 60% (based on the *L*H available). Analysis calculated for C_42_H_48_Cl_2_CuN_8_O_10_ [**4**·2(1-PrOH)] (found values in parentheses): C 52.58 (52.30), H 5.04 (4.79), N 11.68 (11.79)%. Selected IR bands (KBr, cm^−1^): 3308*m*, 3142*m*, 3022*w*, 1616*w*, 1590*m*, 1488*s*, 1452*m*, 1424*w*, 1274*w*, 1162*s*, 1126*s*, 1088*s*, 966*m*, 952*m*, 836*m*, 760*s*, 692*s*, 624*s*, 510*w*, 446*w*.

#### 2.1.5. [Cu(ClO_4_)_2_(*L*H)_4_]·2(2-PrOH) [5·2(2-PrOH)]

A solution of *L*′H (0.180 g, 1.25 mmol) and Cu(ClO_4_)_2_·6H_2_O (0.185 g, 0.5 mmol) in 2-PrOH (25 ml) was stirred for 30 min. The resultant turquoise solution was filtered and then layered with 50 ml of *n*-hexane/Et_2_O (1:2 *v*/*v* ratio) to produce light-purple prismatic crystals after two days. The crystals were collected by filtration and dried in a vacuum desiccator over anhydrous CaCl_2_; the yield was 55% (based on the *L*H available). Analysis calculated for C_36_H_32_Cl_2_CuN_8_O_8_ (**5**) (found values in parentheses): C 51.53 (51.28), H 3.84 (3.60), N 13.35 (13.51)%. Selected IR bands (KBr, cm^−1^): 3320*m*, 3132*s*, 3017*w*, 1615*w*, 1593*m*, 1496*m*, 1458*w*, 1421*w*, 1361*w*, 1274*w*, 1160*s*, 1229*w*, 1117*s*, 1093*s*, 964*m*, 949*m*, 838*m*, 758*s*, 696*m*, 649*m*, 624*s*, 511*w*, 450*w*.

#### 2.1.6. [Cu(ClO_4_)_2_(*L*H)_4_]·2(2-BuOH) [6·2(2-BuOH)]

A solution of *L*′H (0.058 g, 0.4 mmol) and Cu(ClO_4_)_2_·6H_2_O (0.037 g, 0.1 mmol) in 2-BuOH (25 ml) was stirred for 30 min. The resultant turquoise solution was filtered and then layered with 50 ml of *n*-hexane (1:2 *v*/*v* ratio) to produce purple prismatic crystals after four days; the crystals were collected by filtration and dried in a vacuum desiccator over anhydrous CaCl_2_. The overall yield was 30% (based on Cu^II^). Analysis calculated for C_44_H_52_Cl_2_CuN_8_O_10_ [**6**·2(2-BuOH)] (found values in parentheses): C 53.52 (53.25), H 5.31 (5.41), N 11.35 (11.50)%. Selected IR bands (KBr, cm^−1^): 3410*mb*, 3318*m*, 3150*s*, 3027*w*, 1608*w*, 1587*w*, 1496*m*, 1451*w*, 1419*w*, 1353*w*, 1277*w*, 1233*w*, 1120*s*, 1086*s*, 964*m*, 952*m*, 837*m*, 764*s*, 692*m*, 651*m*.

#### 2.1.7. [Cu(ClO_4_)_2_(*L*H)_4_]·2DMF (7·2DMF)

*L*′H (0.173 g, 1.2 mmol) and Cu(ClO_4_)_2_·6H_2_O (0.111 g, 0.3 mmol) were dissolved in a mixture of CH_2_Cl_2_ (25 ml) and DMF (2 ml), and stirred for 30 min. The resultant blue solution was filtered and then layered with 50 ml of *n*-hexane (1:2 *v*/*v* ratio) to produce purple prismatic crystals after two days; the crystals were collected by filtration and dried in a vacuum desiccator over anhydrous CaCl_2_. The overall yield was 30%. Analysis calculated for C_42_H_46_Cl_2_CuN_10_O_10_ (**7**·2DMF) (found values in parentheses): C 51.20 (51.50), H 4.71 (4.48), N 14.22 (14.39)%. Selected IR bands (KBr, cm^−1^): 3151*m*, 3027*w*, 1660*s*, 1609*w*, 1588*m*, 1496*s*, 1441*m*, 1422*w*, 1360*w*, 1254*w*, 1221*w*, 1176*w*, 1130*s*, 1086*s*, 1058*m*, 977*w*, 968*m*, 952*m*, 833*m*, 777*m*, 757*s*, 688*s*, 628*s*, 508*w*, 453*w*.

#### 2.1.8. [Cu(ClO_4_)_2_(*L*H)_4_]·2Me_2_CO (8·2Me_2_CO)

A solution of *L*′H (0.173 g, 1.2 mmol) and Cu(ClO_4_)_2_·6H_2_O (0.111 g, 0.3 mmol) in Me_2_CO (25 ml) was stirred for 30 min. The resultant blue solution was filtered and then layered with 50 ml of *n*-hexane (1:2 *v*/*v* ratio) to produce purple prismatic crystals after one day; the crystals were collected by filtration and dried in air. The yield was 75% (based on Cu^II^). Analysis calculated for C_36_H_32_Cl_2_CuN_8_O_8_ (**8**) (found values in parentheses): C 51.53 (51.80), H 3.84 (3.71), N 13.35 (13.11)%. Selected IR bands (KBr, cm^−1^): 3146*m*, 3022*w*, 1614*w*, 1590*m*, 1492*s*, 1444*m*, 1424*w*, 1364*w*, 1256*w*, 1224*w*, 1176*w*, 1126*s*, 1088*s*, 1055*m*, 980*w*, 966*m*, 954*m*, 836*m*, 774*m*, 760*s*, 692*s*, 626*s*, 510*w*, 448*w*.

#### 2.1.9. [Cu(ClO_4_)_2_(*L*H)_4_]·2THF (9·2THF)

A solution of *L*′H (0.173 g, 1.2 mmol) and Cu(ClO_4_)_2_·6H_2_O (0.111 g, 0.3 mmol) in THF (25 ml) was stirred for 30 min. The resultant turquoise solution was filtered and then layered with 50 ml of Et_2_O (1:2 *v*/*v* ratio) to produce pink prismatic crystals after three days; the crystals were collected by filtration and dried in a vacuum desiccator over silica gel. The yield was 35% (based on Cu^II^). Analysis calculated for C_36_H_32_Cl_2_CuN_8_O_8_ (**9**) (found values in parentheses): C 51.53 (51.84), H 3.84 (3.97), N 13.35 (13.06)%. Selected IR bands (KBr, cm^−1^): 3150*m*, 3026*w*, 1611*w*, 1588*m*, 1494*s*, 1442*m*, 1420*w*, 1361*w*, 1258*w*, 1222*w*, 1174*w*, 1122*s*, 1089*s*, 1058*m*, 977*w*, 965*m*, 952*m*, 833*m*, 774*m*, 758*s*, 690*s*, 625*s*, 512*w*, 446*w*.

#### 2.1.10. [Cu(ClO_4_)_2_(*L*H)_4_]·2(1,4-dioxane) [10·2(1,4-dioxane)]

A solution of *L*′H (0.173 g, 1.2 mmol) and Cu(ClO_4_)_2_·6H_2_O (0.111 g, 0.3 mmol) in 1,4-dioxane (25 ml) was stirred for 30 min. The resultant turquoise solution was filtered and then layered with 50 ml of Et_2_O (1:2 *v*/*v* ratio) to produce pink prismatic crystals after three days; the crystals were collected by filtration and dried in a vacuum desiccator over silica gel. The yield was 15% [based on Cu^II^]. Analysis calculated for C_44_H_48_Cl_2_CuN_8_O_12_ [**10**·2(1,4-dioxane)] (found values in parentheses): C 52.05 (51.68), H 4.76 (4.50), N 11.04 (11.32)%. Selected IR bands (KBr, cm^−1^): 3146*m*, 3028*w*, 1608*w*, 1589*m*, 1491*s*, 1445*m*, 1421*w*, 1358*w*, 1257*w*, 1218*w*, 1177*w*, 1124*s*, 1091*s*, 1057*m*, 980*w*, 963*m*, 950*w*, 830*m*, 772*m*, 761*s*, 688*s*, 626*s*, 512*w*, 445*w*.

#### 2.1.11. [Cu(ClO_4_)_2_(*L*H)_4_]·2EtOAc (11·2EtOAc)

A solution of *L*′H (0.180 g, 1.25 mmol) and Cu(ClO_4_)_2_·6H_2_O (0.185 g, 0.5 mmol) in EtOAc (25 ml) was stirred for 30 min. The resultant turquoise solution was filtered and then stored at low temperature (5°C) to produce orange prismatic crystals after 15 days; the crystals were collected by filtration and dried in a vacuum desiccator over anhydrous CaCl_2_. The yield was 25% (based on Cu^II^). Analysis calculated for C_36_H_32_Cl_2_CuN_8_O_8_ (**11**) (found values in parentheses): C 51.53 (51.79), H 3.84 (3.70), N 13.35 (13.19)%. Selected IR bands (KBr, cm^−1^): 3146*m*, 3022*w*, 2980*m*, 1612*w*, 1577*m*, 1490*s*, 1448*m*, 1424*w*, 1364*w*, 1250*w*, 1168*w*, 1126*s*, 1094*s*, 1055*m*, 980*w*, 968*m*, 954*m*, 912*w*, 838*m*, 774*m*, 762*s*, 692*s*, 626*s*, 508*w*, 452*w*.

#### 2.1.12. [Cu(ClO_4_)_2_(*L*H)_4_]·Et_2_O (12·Et_2_O)

A solution of *L*′H (0.173 g, 1.2 mmol) and Cu(ClO_4_)_2_·6H_2_O (0.111 g, 0.3 mmol) in MeCN (25 ml) was stirred for 30 min. The resultant blue solution was filtered and then layered with 50 ml of Et_2_O (1:2 *v*/*v* ratio) to produce blue blocks after three days; the crystals were collected by filtration and dried in a vacuum desiccator over silica gel. The yield was 40% (based on Cu^II^). Analysis calculated for C_36_H_32_Cl_2_CuN_8_O_8_ (**12**) (found values in parentheses): C 51.53 (51.76), H 3.84 (3.72), N 13.35 (13.20)%. Selected IR bands (KBr, cm^−1^): 3148*m*, 3022*w*, 1616*w*, 1590*w*, 1488*s*, 1445*m*, 1428*w*, 1364*w*, 1256*w*, 1224*w*, 1188*w*, 1126*s*, 1104*m*, 1088*s*, 1055*m*, 980*w*, 966*m*, 954*m*, 902*w*, 834*m*, 774*m*, 760*s*, 692*s*, 626*s*, 510*w*, 446*w*.

### 2.2. Crystallography

Suitable single crystals of the compounds, coated with paratone-N oil, were attached in cryo-loops at the end of a copper pin. Diffraction data were collected by the ω-scan technique on a Rigaku Oxford Diffraction SuperNova diffractometer under a stream of nitro­gen gas at 100 (2) K using Mo *K*α radiation (λ = 0.7107 Å), except for compound **6**·2(2-BuOH) where Cu *K*α radiation (λ = 1.5418 Å) was used. Data were collected and processed by the *CrysAlis CCD* and *CrysAlis RED* (Rigaku Oxford Diffraction, 2015 ▸) software, respectively ; an empirical absorption correction using spherical harmonics, implemented in the SCALE3 ABSPACK scaling algorithm, was applied to the intensities of the collected reflections. The structures were solved using direct methods with *SIR92* (Altomare *et al.*, 1994 ▸) and *SHELXT* (Sheldrick, 2015*a* ▸) and refined by full-matrix least-squares on *F*^2^ with *SHELXL-2014/7* (Sheldrick, 2015*b* ▸). All non-H atoms were refined anisotropically; carbon-bound H atoms were included in calculated positions (riding model). All imidazole H atoms on the pyrrolic-type N1 atom of the ligands, together with the H atoms of the solvent water molecule O5 in **1**·3.3(H_2_O) and the hydroxyl H atoms of the methanol, ethanol, 1-propanol and 2-butanol solvent molecules in compounds **2**·2MeOH, **3**·2EtOH, **4**·2(1-PrOH) and **6**·2(2-BuOH), respectively, were located in difference Fourier maps and refined isotropically applying soft distance restraints (*DFIX*); however, the water O6*A*/O6*B* solvent molecule in **1**·3.3(H_2_O) was disordered and its hydrogen atoms could not be reliably located. Non-routine aspects of structure refinement are as follows: a phenyl ring of the ligands in complexes **2**·2MeOH, **3**·2EtOH and **12**·Et_2_O is positionally disordered and has been modelled over two sites (domain ratio: 65:35, 55:45 and 50:50, respectively); the coordinated perchlorate ions in complexes **2**·2MeOH and **3**·2EtOH are similarly disordered (70:30 and 56:46, respectively); disorder also affects the O6*A*/O6*B* water molecule in **1**·3.3(H_2_O), the MeOH solvent molecule in **2**·2MeOH (65:35), the EtOH solvent in **3**·2EtOH (50:50) and the di­ethyl ether solvent in **12**·Et_2_O (orientational disorder about a centre of symmetry). Geometric/crystallographic calculations were carried out using *PLATON* (Spek, 2009 ▸), *OLEX2* (Dolomanov, 2009 ▸), and *WinGX* (Farrugia, 2012 ▸) packages; molecular/packing graphics were prepared with *Mercury* (Macrae *et al.*, 2020 ▸). Experimental details are listed in Table 1 ▸.

## 3. Results and discussion

### 3.1. Comments on synthesis

Various reactions were initially explored, aimed at preparing the largest possible number of Cu^II^ complexes of the *L*′H ligand; our efforts included differing reagent ratios and concentrations, solvents, temperatures and crystallization conditions. It was soon identified that the reaction system yielded solvatomorphic compounds with the general form [Cu(ClO_4_)_2_(*L*H)_4_]·*x*(solvent). Utilizing a variety of crystallization solvents (polar protic, polar aprotic and non-polar) a series of 12 crystalline solvatomorphs have been isolated (Fig. 1 ▸). Copper(II) is air stable and the synthetic work was thus performed under aerobic conditions in the normal laboratory atmosphere. The compounds were obtained by reactions with a metal-to-ligand ratio of 1:4 or 1:2.5 and have an octahedral geometry for the metal centre; their colour is blue. The complexes are insoluble in Et_2_O, *n*-hexane, CH_2_Cl_2_ and CHCl_3_, moderately soluble in alcohols, and readily soluble in DMF and DMSO. The very good solubility in the latter two solvents might indicate reactions (and therefore decomposition) of the complexes with the solvents which are strong donors, probably replacing the perchlorato groups and/or *L*H ligands from the coordination sphere of Cu^II^. Crystals grown with 1-butanol as well as MeNO_2_ solvents were of poor diffraction quality. Attempts with other solvents such as MeCN, CH_2_Cl_2_, CHCl_3_, DMSO and pyridine did not produce the anticipated solvatomorphs. Crystals of the solvatomorph **5** with 2-propanol, [Cu(ClO_4_)_2_(*L*H)_4_]·2(2-propanol), were also grown and its crystal structure was unambiguously determined allowing a description of the intermolecular association between the complex molecules and the solvent; however, due to its poor refinement indices (*R*_1_ = 13.9%), the structure has not been deposited with the CCDC database and not listed in Tables 1 ▸ and 2 ▸. Moreover, the crystal structure is not discussed. As mentioned in the caption of Fig. 1 ▸, the initially used 4-phenyl­imidazole (*L*′H) has been transformed to its 5-phenyl­imidazole (*L*H) when coordinated to copper(II) in the present complexes. Tautomerism occurs when two (or more) constitutional isomers of different connectivity exist in a dynamic equilibrium with each other. The most often observed case of tautomerism is the prototropic tautomerism which involves a transfer of a proton from one position in a molecule to another. For cyclic compounds, the H atom can be exchanged between ring atoms (annular tautomerism) or between a ring and side-chain atoms (side-chain tautomerism). Thus, the *L*′H → *L*H transformation observed in complexes **1**−**12** is annular tautomerism. Two polymorphs of *L*′H have been characterized by single-crystal X-ray crystallography (Claramunt *et al.*, 2002 ▸). Solid state ^13^C and ^15^N NMR studies have revealed that *L*′H is the only tautomer that exists as free compound in the solid state. In solution, both tautomers exist. Since the complexes were formed in solution, it appears that *L*H is the thermodynamically stable tautomer when it is coordinated to copper(II). A possible reason for the observed preference is the position of the donor atom, *i.e.* N3. This would be close enough to the phenyl ring in complexes of *L*′H creating a steric hindrance, but far from the phenyl ring in the 5-tautomer (*L*H) relieving the hindrance. The preference should also be associated with subtle supramolecular characteristics in the crystal structures of the copper(II) complexes of *L*H and especially with the strength of the hydrogen bonds. Various trials to obtain crystals of the solvent-free form (including solvothermal reactions, tri­ethyl orthoformate as a drying agent, and solvent mixtures) were in vain. The crystalline products were characterized by IR spectroscopy, microanalyses, single-crystal X-ray diffraction and TGA experiments for selected compounds. It should be mentioned at this point that some complexes, *i.e.***2**·2MeOH, **3**·2EtOH, **5**·2(2-PrOH), **8**·2Me_2_CO, **9**·2THF, **11**·2EtOAc and **12**·Et_2_O, were analysed as lattice solvent-free (**2**, **3**, **5**, **8**, **9**, **11** and **12**), while for the rest, the crystallographic formula appears the same as the analytical formula (as obtained from C, H and N microanalyses).

### 3.2. Description of the structures

**Figure 2:**
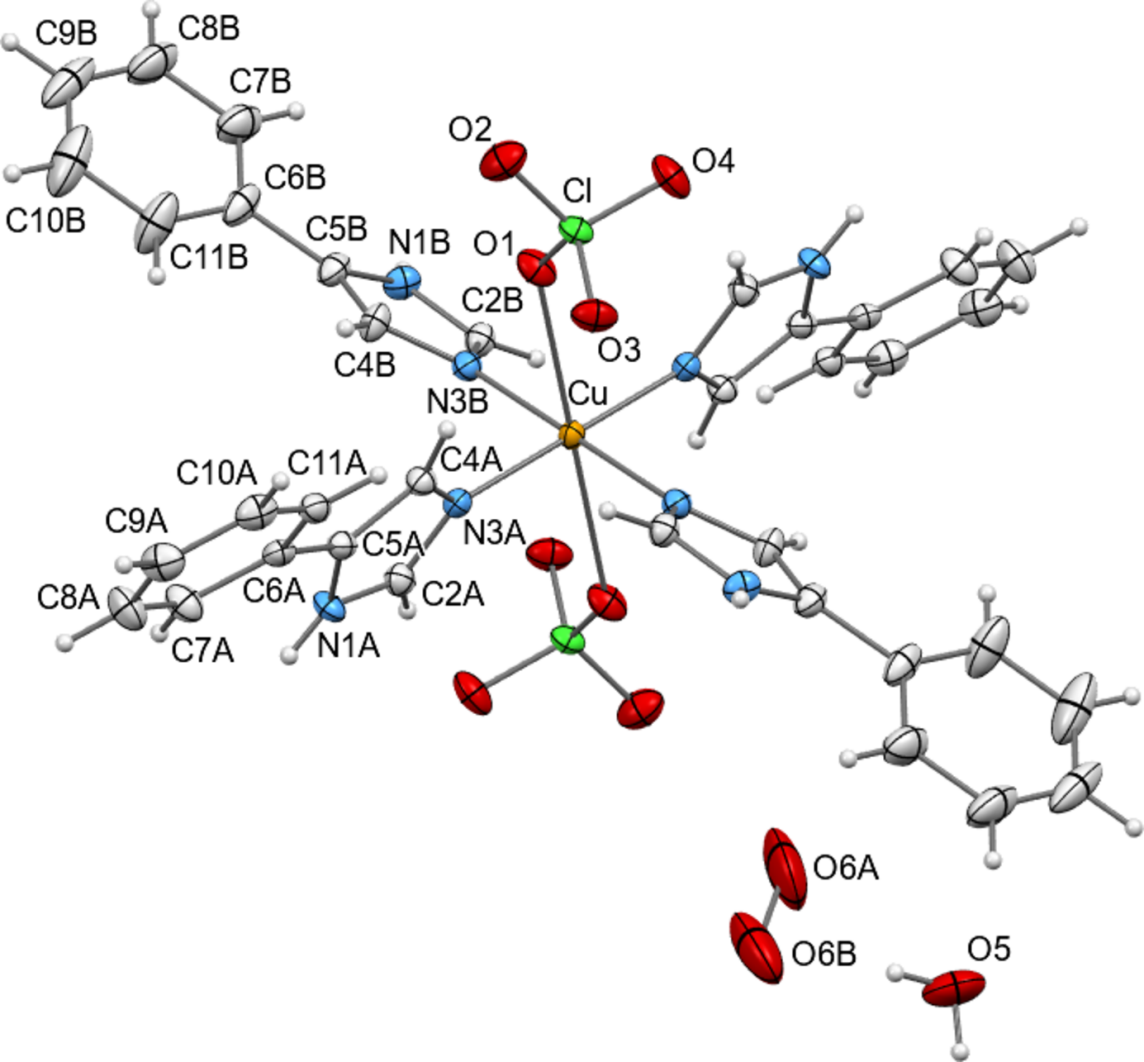
A view of the structure of solvatomorph **1**·3.3(H_2_O). Displacement ellipsoids are drawn at the 50% probability level. Atoms generated by symmetry through the inversion centre located on the copper atom are not labelled. Both orientations of the disordered water solvent molecule O6 are shown.

To facilitate discussion and comparison, the exact same numbering scheme has been assigned (where applicable) to the *L*H ligand atoms and the coordinated perchlorate ions for all compounds presented herein (Fig. 2 ▸ and S1–S10).

The compounds crystallize in the space group 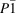, with the exception of **9**·2THF which crystallizes in *P*2_1_/*n* (Table 1 ▸). The asymmetric unit of all compounds contains half a [Cu(ClO_4_)_2_(*L*H)_4_] host complex (*Z*′ = ½), with the copper(II) atom situated on an inversion centre. Each copper centre has a slightly distorted octahedral N_4_O_2_ coordination involving a pyridine-type nitro­gen atom from each ligand in the equatorial positions and an oxygen atom from each terminal perchlorate in the axial positions. The Cu^II^—O_perchlorato_ bonds are weak (2.49–2.73 Å) and thus the metal centre is in an axially elongated octahedral environment (4+2); this is a consequence of the Jahn–Teller effect, typical for 3*d*^9^ systems. The bond lengths and angles observed for the current complexes are similar. To compare the conformation of the [Cu(ClO_4_)_2_(*L*H)_4_] complexes in the 12 solvatomorphs, Table 2 ▸ lists the relative orientation of the imidazole rings within each complex, as well as the orientation of the phenyl ring relative to the imidazole ring in each *L*H ligand. It can be seen that the conformation of the complexes is similar, with some differences that presumably help to minimize steric hindrance among the ligands, accommodate the incorporated solvents (taking into account their size and molecular shape) and allow them to engage in strong hydrogen-bonding patterns. The deviation in the orientation between the imidazole rings *A* and *B* in the complexes is 17.1° (89.6–72.5°), while that between the phenyl rings and the imidazole to which each ring is attached is, as expected, higher: 17.4° (20.1–2.7°) for ligands *A*, and 28.6° (35.1–6.5°) for ligands *B*. We could postulate that since the phenyl rings are further away from the core and are not engaged as strong synthons, they are more flexible to orient themselves and accommodate better within the crystal space. In view of the similarities in the space group, the unit-cell dimensions/volumes and the conformation of the [Cu(ClO_4_)_2_(*L*H)_4_] host complexes, the 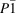 compounds can be described as isostructural (Bombicz, 2024 ▸). However, the different hydrogen-bonding capabilities of the solvent molecules lead to variations in the structurally significant intermolecular interactions (*see below*).

The observed solvatomorphs were sorted into three categories on the basis of the type of solvent incorporated and the subsequent hydrogen-bonding patterns formed. There are two solvent molecules per centrosymmetric [Cu(ClO_4_)_2_(*L*H)_4_] complex, with the exception of the hydrate **1** (3.3 disordered water molecules) and the solvate **12** (one di­ethyl ether molecule lying on an inversion centre).

#### 3.2.1. Polar protic solvents: compounds 1–6

**Figure 3:**
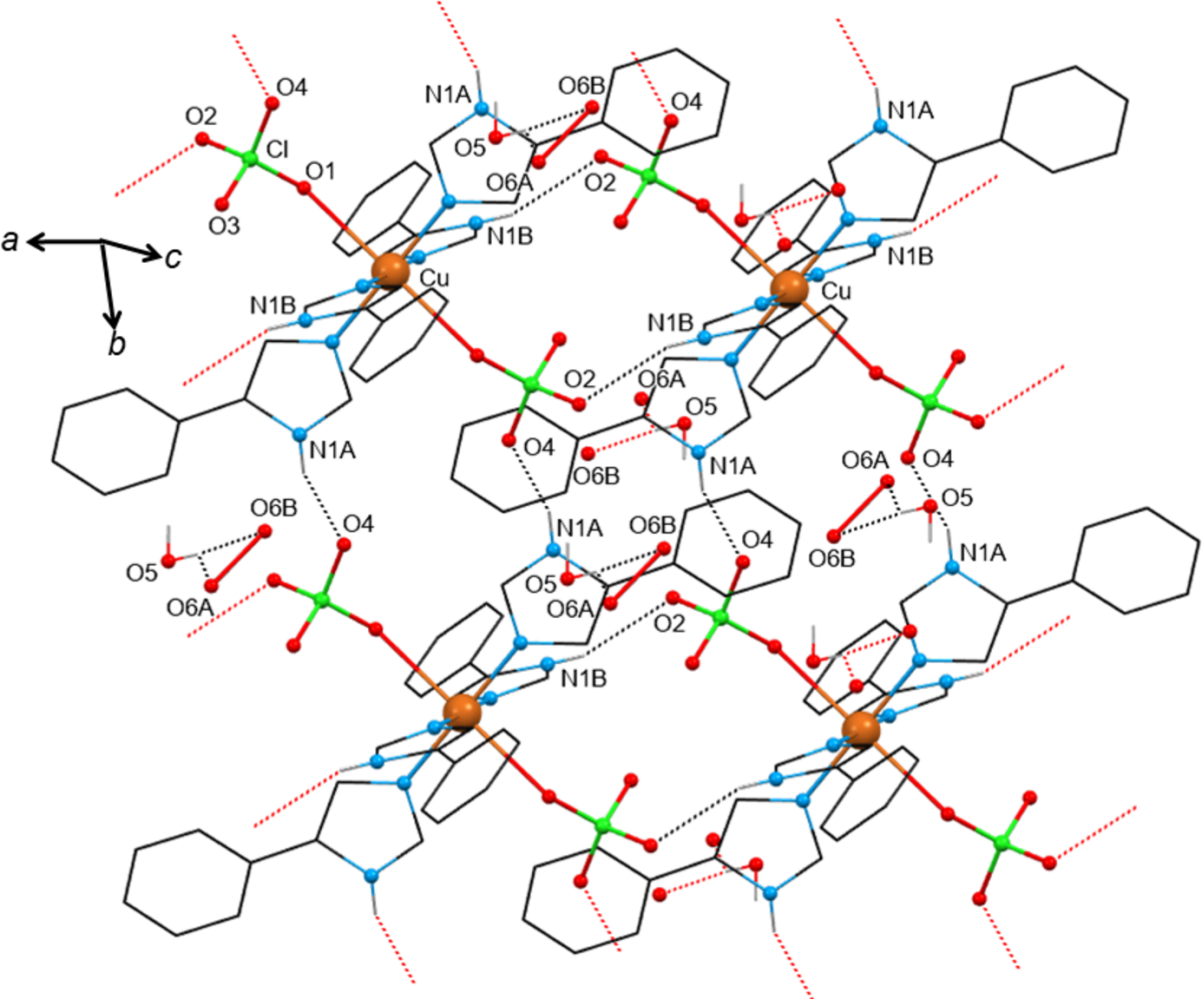
View of a 2D layer of the crystal structure of solvatomorph **1**·3.3(H_2_O) organized by hydrogen-bonding motifs. The water molecules are labelled as O5 and O6*A*/O6*B*. Hanging contacts are represented by dotted lines in red. Only the H atoms involved in strong intermolecular interactions are drawn. Atoms generated by symmetry operations have been included, but have been labelled identically to the asymmetric unit.

The solvents contained in these compounds are water (**1**), methanol (MeOH) (**2**), ethanol (EtOH) (**3**), 1-propanol (1-PrOH) (**4**), 2-propanol (2-PrOH) (**5**) and 2-butanol (2-BuOH) (**6**). These solvents have an effective donor–acceptor capability and are therefore expected to participate in multi-point recognition, *e.g.* in solute–solvent hydrogen bonding. At the same time, the [Cu(ClO_4_)_2_(*L*H)_4_] molecule itself possesses classic hydrogen-bond donors (the imidazole N—H group) and acceptors (the perchlorate oxygen atoms) which in turn could be involved in solute–solute bonding. In all, except in the water-containing compound **1**, the packing organization is the same. Packing diagrams of compounds **1**·3.3(H_2_O) and **2**·2MeOH are shown in Figs. 3 ▸ and 4 ▸, respectively, and that of compound **6**·2(2-BuOH) in Fig. S11. The main hydrogen-bonding motifs in the compounds are listed in Table 3 ▸. The [Cu(ClO_4_)_2_(*L*H)_4_] molecules are directly linked *via* robust N1*A*—H1*A*⋯O4_perchlorate_ synthons along the *b* axis. The second donor group N1*B*—H1*B* of the complex is bridged to another perchlorate by the incorporated alcohol along the *a* axis. The solvents act both as donors and as acceptors forming doubly hydrogen-bonded synthons N1*B*—H1*B*⋯O5_alcohol_—H⋯O2_perchlorate_. Thus, the linked molecules form rigid 2D layers parallel with the *ab* plane, with the solvents situated on either side of the layers. In the hydrated compound **1**, the water molecules are highly disordered, they occupy almost the same position in the unit cell as the solvents in compounds **2**–**6**, but do not bridge neighbouring [Cu(ClO_4_)_2_(*L*H)_4_] complexes. Instead, the N1*B*—H1*B* donor and the O2_perchlorate_ acceptor are directly linked through the N1*B*—H1*B*⋯O2_perchlorate_ interaction, compensating for the absence of the bridging solvent by proper rotations of the imidazole and perchlorate groups involved. There is a notable number of aromatic rings in the complexes; however, only a few π⋯π interactions have been identified in their crystal structures (Table S1) between rings of adjacent layers contributing to the structure extension and stabilization in 3D. It seems that these weak interactions only occur when they are sterically allowed and certainly not at the expense of the structure-directing synthons which are fully exploited.

#### 3.2.2. Polar aprotic solvents: compounds 7–10

The solvents involved in this group are di­methyl­formamide (DMF) (**7**), acetone (Me_2_CO) (**8**), tetra­hydro­furan (THF) (**9**) and 1,4-dioxane (**10**), featuring only hydrogen-bond acceptors. The supramolecular self-assembly in **7**, **8** and **9** is based on the same pattern observed in compounds **2**−**6**: the [Cu(ClO_4_)_2_(*L*H)_4_] molecules are linked *via* N1*A*—H1*A*⋯O4_perchlorate_ synthons forming 1D tapes along the *b* axis (for **7** and **8**) and along the *c* axis (for **9** in the space group *P*2_1_/*n*). The solvents are only terminally hydrogen bonded to the second donor group of the structure, namely N1*B*—H1*B*⋯O5_solvent_. A view of the packing of compound **7**·2DMF is shown in Fig. 5 ▸ and those of **8**·2Me_2_CO and **9**·2THF in Fig. S12. The pattern of π⋯π interactions observed in compounds **1**–**6** is also present in **7**–**9** (Table S1). However, some of these interactions are disrupted in compound **9** due to the interference of THF solvents between aromatic rings. In compound **10** the asymmetric unit contains two halves of 1,4-dioxane solvent molecules and the supramolecular arrangement is somewhat different. Due to their special position within the unit cell, the two dioxane molecules are arranged alternately in channels along the *b*-axis direction [Fig. 6 ▸(*a*)]. As in all previous structures **1**–**9**, the [Cu(ClO_4_)_2_(*L*H)_4_] molecules are directly linked *via* the N1*A*—H1*A*⋯O4_perchlorate_ pattern along the *b* axis. The first 1,4-dioxane molecule (O5) is doubly hydrogen bonded, *via* its two centrosymmetrically related oxygen atoms (O5 and O5*), connecting two neighbouring [Cu(ClO_4_)_2_(*L*H)_4_] molecules in the [101] direction: N1*B*—H1*B*⋯O5–//–O5*⋯H1B*—N1B*. Thus, the structure is organized in 2D layers parallel with the 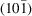 plane [Fig. 6 ▸(*b*)]. Some weak π⋯π interactions between these layers help to organize the structure into a 3D assembly. Similar to compound **9**, the positioning of the 1,4-dioxane molecules within the layers prevents the formation of some π⋯π interactions as was the case in some of the previously discussed structures **1**–**8**. The second 1,4-dioxane molecule (O6) is not involved in any major bonding pattern, except for two weak C12—H12*B*⋯O6 interactions with the first dioxane molecule (O5) on either side and running within the solvent channel along the *b* axis.

#### 3.2.3. Non-polar solvents: compounds 11 and 12

The solvatomorph with ethyl acetate (EtOAc) (compound **11**) has been included in this category; EtOAc is commonly referred to as a non-polar to weak polar aprotic solvent. The supramolecular self-assembly in **11** (Fig. S13) is based on the same pattern as in compounds **7** and **8**. Namely, the [Cu(ClO_4_)_2_(*L*H)_4_] molecules are linked *via* N1*A*—H1*A*⋯O4_perchlorate_ synthons forming 1D tapes and the ethyl acetate solvents are only terminally hydrogen bonded through their carbonyl oxygen atom to the second donor group of the structure: N1*B*—H1*B*⋯O5_solvent_. However, the 1D tapes spread along the *a* axis, not *b* as in compounds **7** and **8**. All aromatic rings of the [Cu(ClO_4_)_2_(*L*H)_4_] molecule are involved in π⋯π interactions (Table S1), adding to the stability of the resulting 3D structure. There is one di­ethyl ether (Et_2_O) molecule in the unit cell of compound **12** lying on an inversion centre (Fig. 7 ▸). The Et_2_O molecule links two adjacent [Cu(ClO_4_)_2_(*L*H)_4_] units *via* weak *sp*^3^ C—H⋯O_perchlorate_ interactions, but it is not involved in any hydrogen-bonding motif. Consequently, the structure-organizing pattern is based on synthons between the donor and acceptor groups of the [Cu(ClO_4_)_2_(*L*H)_4_] complex itself, the same as those observed in the structure of solvatomorph **1**, in which the disordered water solvents do not participate in the hydrogen-bonding scheme. This means that the [Cu(ClO_4_)_2_(*L*H)_4_] complexes are linked *via* strong hydrogen bonds between the N1*A*—H1*A* and N1*B*—H1*B* donor groups and the perchlorate ions of neighbouring complexes along *b* and *a* axes, respectively, thus forming 2D layers parallel with the *ab* plane. As with the previous structures, some weak π⋯π interactions between aromatic rings of adjacent layers help to extend and stabilize the structure in 3D (Table S1).

A number of weak C—H⋯O intra- and intermolecular contacts that enhance the stability of the supramolecular assembly are also present in the 12 structures discussed. A few C—H⋯π interactions were also observed for each structure and a complete list of these, with the relevant geometrical parameters, is presented in Table S2. These interactions (Nishio *et al.*, 1998 ▸, 2009 ▸) are the weakest hydrogen bonds that operate between a soft acid CH and a soft or intermediate base π system and it has been recognized that this type of weak interaction plays a role in crystal packing, host–guest chemistry, self-assembly and chiral recognition. As can be seen in Table S2, most of these interactions are of the C(aromatic)—H⋯π type, along with a few C(*sp*^3^)—H⋯π (originating from the methyl/methyl­ene groups of the solvents).

#### 3.2.4. Location of the solvents in the crystal structures

**Figure 4:**
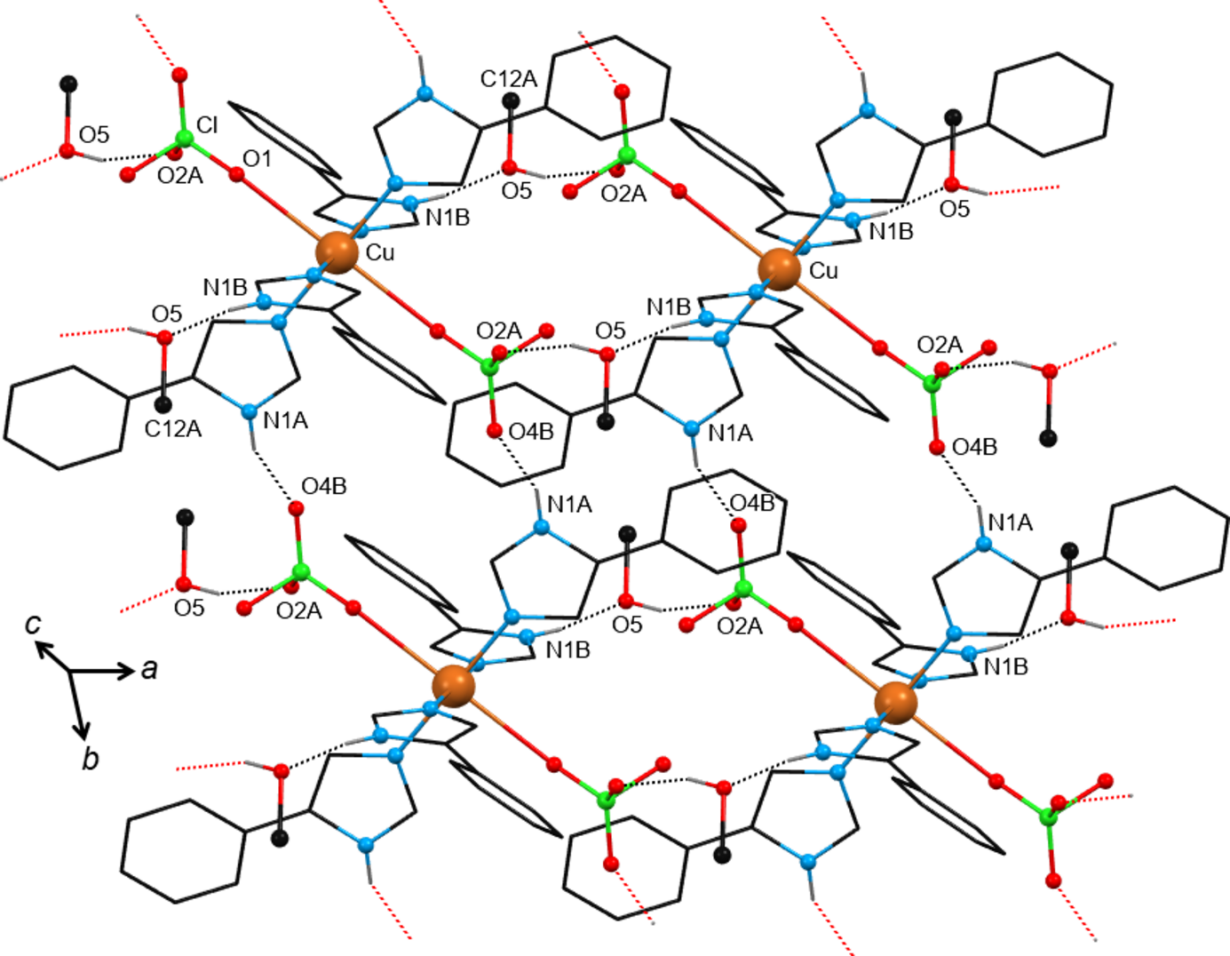
View of a 2D layer of the crystal structure of solvatomorph **2**·2MeOH organized by hydrogen-bonding motifs. The atoms of the methanol molecule are labelled as O5 and C12*A*. Only the H atoms involved in strong intermolecular interactions are drawn. For clarity, only the major components of the disordered groups of the structure are shown. The MeOH molecules are directed along the *a* axis of the unit cell. Hanging contacts are represented by dotted lines in red. Atoms generated by symmetry operations have been included, but have been labelled identically to the asymmetric unit.

**Figure 5:**
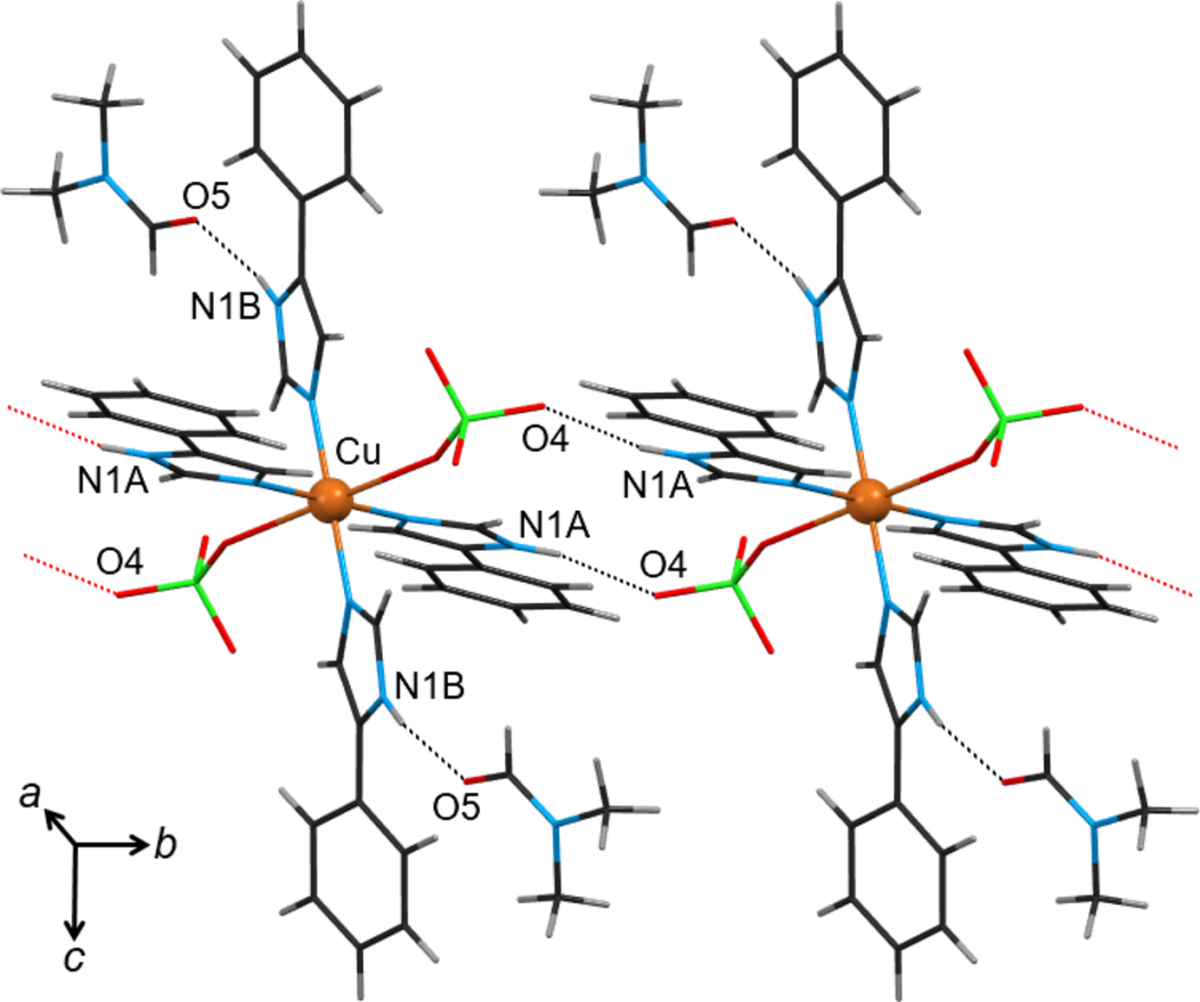
1D tape in the crystal structure of solvatomorph **7**·2DMF formed by the [Cu(ClO_4_)_2_(*L*H)_4_] molecules *via* N1*A*—H1*A*⋯O4_perchlorate_ synthons along the *b* axis. The DMF solvents are only terminally hydrogen-bonded to the host complexes. Atoms generated by symmetry operations have been included, but have been labelled identically to the asymmetric unit.

**Figure 6:**
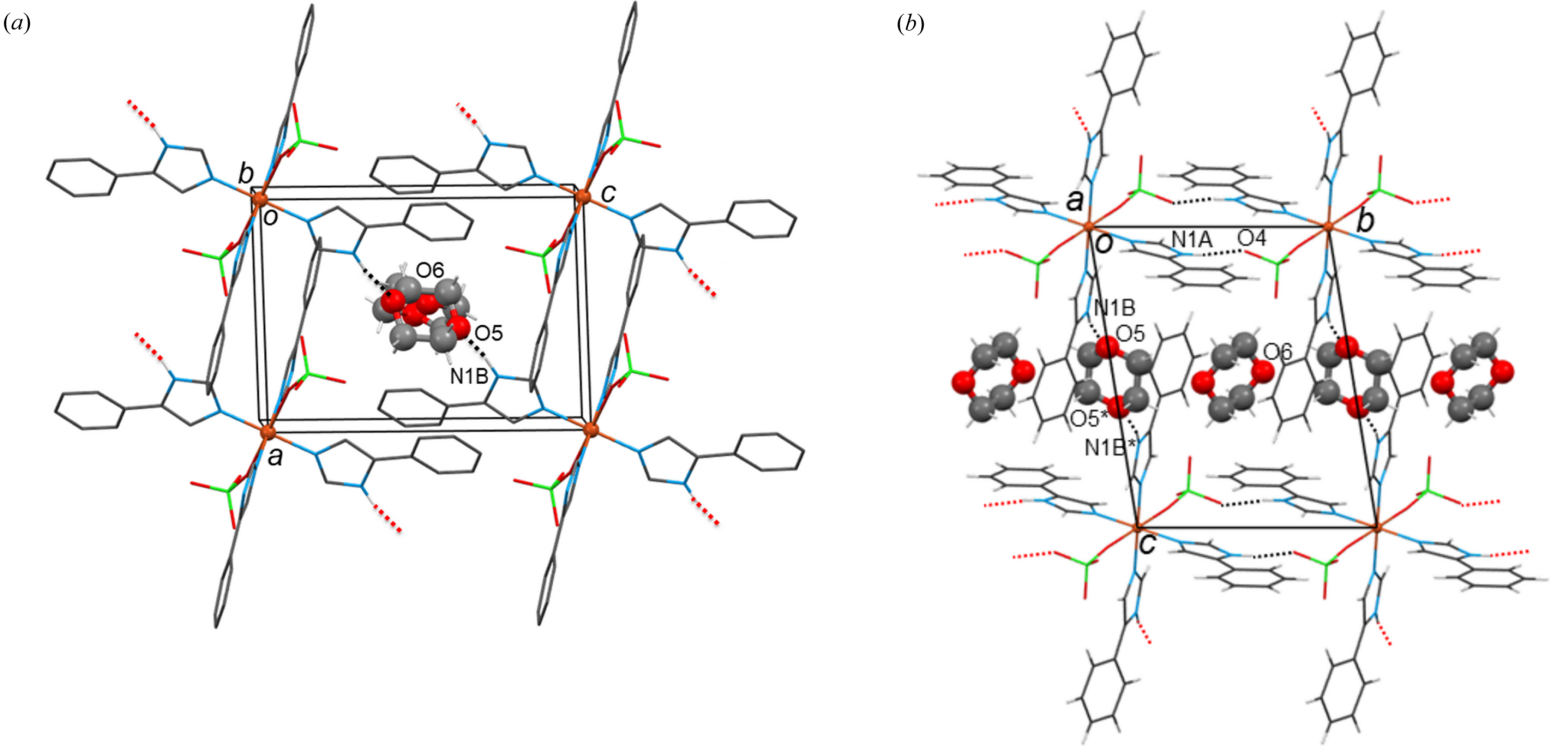
(*a*) The dioxane molecules in compound **10**·2(1,4-dioxane) filling the channels along the *b* axis in the middle of the unit cell. Only the H atoms involved in the interactions are shown. (*b*) The 2D layer formed by N1*A*—H1*A*⋯O4 interactions between the *L*H ligands and the perchlorate ions along *b*, and N1*B*—H1*B*⋯O5 interactions between the ligands and the 1,4-dioxane molecules in the [101] direction. Hanging contacts are drawn by red dotted lines. The dioxane molecules are highlighted.

**Figure 7:**
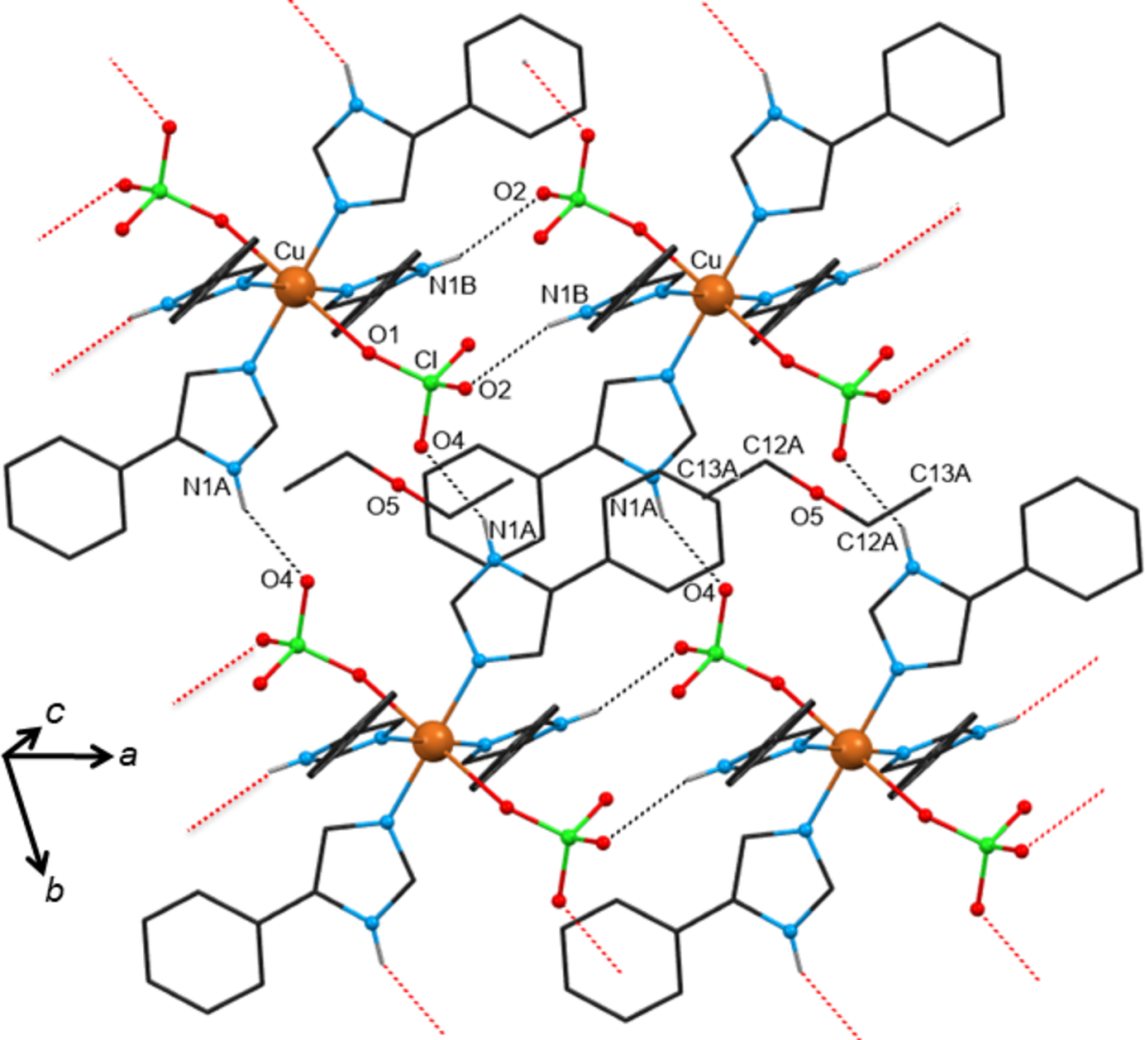
View of a 2D layer of the crystal structure of solvatomorph 1**2**·Et_2_O organized by hydrogen-bonding motifs. For clarity, only one orientation of the disordered groups of the structure is shown. The atoms of the di­ethyl ether solvent are labelled as O5, C12*A* C13*A*. Only the H atoms involved in strong intermolecular interactions are drawn. The di­ethyl ether molecules are directed along the *a* axis of the unit cell. Hanging contacts are represented by dotted lines in red.

**Figure 8:**
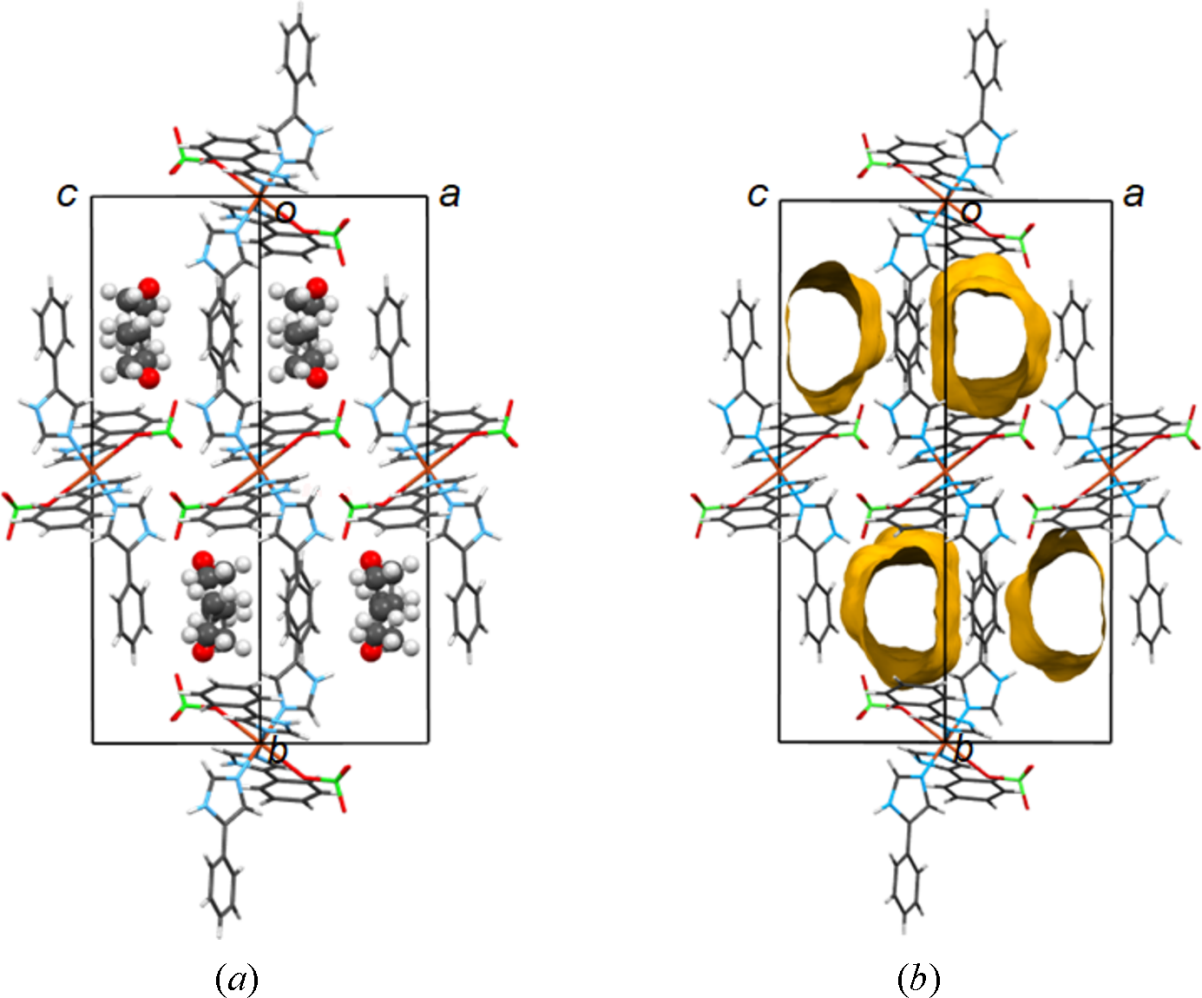
The crystal structure of solvatomorph **9**·2THF showing the solvent channels running parallel to the direction [101]. (*a*) The THF solvent molecules are highlighted. (*b*) The solvents have been removed and the channels containing them are drawn in yellow (VOIDS option in *MERCURY* software; Macrae *et al.*, 2020 ▸).

Inspection of the solvatomorphs shows that the solvent molecules are situated in channels (Gallagher & Mocilac, 2021 ▸) within their crystal structures. Based on *PLATON* analysis, the calculated void space of the channels (after the removal of the solvent molecules of the structures) ranges from 15.9% to 26.9% of the unit-cell volume. That is, 20.7% for **1**·3.3(H_2_O), 18.1% for **2**·2MeOH, 15.9% for **3**·2EtOH, 24.7% for **4**·2(1-PrOH), 26.6% for **6**·2(2-BuOH), 26.9% for **7**·2DMF, 19.7% for **8**·2Me_2_CO, 22.2% for **9**·2THF, 24.4% for **10**·2(1,4-dioxane), 26.6% for **11**·2EtOAc and 16.3% for **12**·Et_2_O (with one Et_2_O molecule in the unit cell). The solvent channels of the structures in 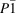 (except **10**) are directed along the *a* axis of the unit cell (*e.g.* see Fig. 4 ▸ for **2**·2MeOH). As previously reported, the asymmetric unit of **10** contains *two* half 1,4-dioxane molecules lying in special positions of 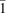 symmetry and this results in somewhat different solvent packing, with the two dioxane molecules arranged alternately in channels along the *b*-axis direction [Fig. 6 ▸(*b*) and S14]. In the solvatomorph **9**·2THF, crystallizing in *P*2_1_/*n*, the solvent channels run parallel to the direction [101] (Fig. 8 ▸).

#### 3.2.5. Brief discussion on TGA and IR results

The TGA patterns of selected complexes (Fig. S15) in the 25–800 °C region indicate similar decomposition schemes. The measurements were performed on freshly (*i.e.* not dried) microcrystalline samples of the complexes. Upon heating the solvent molecules are gradually lost at temperatures up to ∼160 °C depending on the solvent that is present in the compound. Then a plateau appears up to ∼280 °C indicating that the unsolvated complexes are thermally stable below this temperature. Two points deserve a comment here. First, complex **12** is thermally stable because the volatile Et_2_O solvent is readily lost in the air during the grinding of the crystals before the measurement. Second, the experimental mass loss due to the removal of the solvents is lower than the theoretical one. For example, the experimental mass loss for **3**·2EtOH in the 25–145 °C region is 6.9%, while the theoretical one (for the removal of two moles of EtOH per mole of the complex) is 9.9%. This can be attributed to the fact that an amount of solvent has been already lost at room temperature from the microcrystalline powder before the measurement; this observation is reinforced by the fact that the crystals gradually transform to crystalline powders at room temperature (presumably because of a partial solvent loss).

In the IR spectra of the well dried complexes, the medium-intensity bands in the 3320−3100 cm^−1^ range are attributed to the stretching vibration of the N—H bond, *v*(H). In the spectrum of **1**·3.3(H_2_O), the *v*(OH)_water_ vibration appears at ∼3430 cm^−1^. The *v*(OH) bands of 1-PrOH and 2-BuOH appear at ∼ 3415 cm^−1^ in the spectra of the corresponding complexes. The rather low wavenumber and broadness of these bands are indicative of hydrogen bonds which are present in the crystal structures. Such bands do not appear in **2**·2MeOH, **3**·2EtOH and **5**·2(2-PrOH) because the samples used were solvent-free (see Section 2.1). For the same reason, the characteristic *v*(CO) modes of Me_2_CO and EtOAc do not appear in the spectra of **8**·2Me_2_CO and **11**·2EtOAc. In contrast, the spectrum of **7**·2DMF exhibits the characteristic carbonyl stretch, *v*(CO), of DMF because this solvent is retained in the sample after drying and hence when recording the IR spectrum. A characteristic feature of all spectra is the appearance of bands due to the perchlorate ligands. The free ClO_4_^−^ ion belongs to the high-symmetry point group *T*_d_. Of the four vibration fundamentals, only *v*_3_(*F*_2_)[*v*_d_(ClO)] and *v*_4_(*F*_2_)[δ_d_(ClO)] are IR-active. If the symmetry of the ion is lowered by coordination, the degenerate vibrations split and Raman-active modes appear in the IR spectrum. The lowering of symmetry caused by coordination is different for complexes containing monodentate (*C*_3v_) and bidentate (*C*_2v_) perchlorato groups (Nakamoto, 1986 ▸). For the former the *v*_1_(*A*_1_), *v*_2_(*E*), *v*_3_(*A*_1_), *v*_3_(*E*), *v*_4_(*A*_1_) and *v*_4_(*E*) should appear in the IR spectrum. These bands are clearly visible at ∼965, ∼450, ∼1090, ∼1120, ∼645 and ∼625 cm^−1^, respectively. The *v*_3_ bands show signs of further splitting (appearance of a band at ∼950 cm^−1^) which might be an indication of a further symmetry lowering to *C*_2v_. This spectral observation can be explained by the involvement of the uncoordinated perchlorate oxygen atoms in hydrogen bonding which creates a pseudobridging (bidentate or even tridentate) ClO_4_^−^.

## 4. Conclusions

The complex of copper(II) with the 5-phenyl­imidazole/perchlorate ligand system exhibits a remarkable ability to form solvatomorphs and has been crystallized as a solvated form from a large number of polar protic, polar aprotic and non-polar solvents. It includes H_2_O (**1**), MeOH (**2**), EtOH (**3**), 1-PrOH (**4**), 2-PrOH (**5**), 2-BuOH (**6**), DMF (**7**), Me_2_CO (**8**), THF (**9**), 1,4-dioxane (**10**), EtOAc (**11**) and Et_2_O (**12**). There are two solvent molecules per [Cu(ClO_4_)_2_(*L*H)_4_] unit, except for the hydrate **1** (containing 3.3 disordered H_2_O molecules) and the solvate **12** (with one Et_2_O molecule). The complexes are isostructural and crystallize in the space group 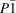 , whereas the THF solvate **9** crystallizes in the space group *P*2_1_/*n*. The [Cu(ClO_4_)_2_(*L*H)_4_] host molecules in the solvatomorphs have similar conformations with small differences in the orientation of the aromatic rings, possibly to comply with steric hindrance, facilitate the formation of hydrogen-bonding synthons and accommodate the various incorporated solvents.

The [Cu(ClO_4_)_2_(*L*H)_4_] complexes form a host framework and the solvent molecules reside in channels along the *a* axis of the unit cell, except for the 1,4-dioxane molecules in **10** which are in special positions and are arranged in channels along the *b* axis, and the THF molecules in **9** (in the *P*2_1_/*n*) which are located in channels along [101]. The calculated void space of the channels, after the removal of the solvent molecules, ranges from 15.9% to 26.9% of the unit-cell volume. That is, solvent molecules containing two to six light non-H atoms (C, O, N) have been found to be readily incorporated into the channels of the complexes’ structure.

All potential hydrogen-bond functionalities of the [Cu(ClO_4_)_2_(*L*H)_4_] units and the solvents are utilized in the course of the crystallization process. Analysis of the crystal packing shows that the supramolecular assembly is directed by strong recurring N—H⋯ motifs among the [Cu(ClO_4_)_2_(*L*H)_4_] host molecules. The solvents are located in channels and, with the exception of the disordered waters in **1** and the di­ethyl ether molecule in **12**, are involved in the formation of hydrogen bonds with the [Cu(ClO_4_)_2_(*L*H)_4_] units, serving as both hydrogen-bond acceptors and donors by bridging the complexes (for the polar protic solvents in **2**–**6**), or solely as hydrogen-bond acceptors terminally linked to the complex molecules (for the polar/non-polar aprotic solvents in **7**–**11**). Since the molecules of the complexes are bridged by the guest solvent molecules (but not only by them), the latter can be considered as having a type of templating behaviour.

Work is in progress in our laboratories to: (*a*) extend this type of chemistry in analogous systems with other, non-Jahn–Teller distorted divalent metals, *e.g.* Mn^II^, Fe^II^, Co^II^, Ni^II^ and Zn^II^ and to understand if the supramolecular characteristics of octahedral [*M*(ClO_4_)_2_(5-phenyl­imidazole)_4_] complexes depend on the metal ion; (*b*) prepare and study Cu^II^ complexes with the 5-phenyl­imidazole/*X*^−^ ‘blends’ where *X*^−^ is the nitrate, tetra­fluoro­borate, trifluoromethanesulfonate and sulfate ion in order to investigate their molecular structures and supramolecular motifs of the products; and (*c*) synthesize 3*d*-metal complexes containing the 4-phenyl­imidazole tautomer (Fig. 1 ▸). Also, we have in mind to support selected experimental data with DFT calculations.

## Supplementary Material

Crystal structure: contains datablock(s) 1, 2, 3, 4, 6, 7, 8, 9, 10, 11, 12. DOI: 10.1107/S2052520624005948/aw5090sup1.cif

Structure factors: contains datablock(s) 1. DOI: 10.1107/S2052520624005948/aw50901sup2.hkl

Structure factors: contains datablock(s) 2. DOI: 10.1107/S2052520624005948/aw50902sup3.hkl

Structure factors: contains datablock(s) 3. DOI: 10.1107/S2052520624005948/aw50903sup4.hkl

Structure factors: contains datablock(s) 4. DOI: 10.1107/S2052520624005948/aw50904sup5.hkl

Structure factors: contains datablock(s) 6. DOI: 10.1107/S2052520624005948/aw50906sup6.hkl

Structure factors: contains datablock(s) 7. DOI: 10.1107/S2052520624005948/aw50907sup7.hkl

Structure factors: contains datablock(s) 8. DOI: 10.1107/S2052520624005948/aw50908sup8.hkl

Structure factors: contains datablock(s) 9. DOI: 10.1107/S2052520624005948/aw50909sup9.hkl

Structure factors: contains datablock(s) 10. DOI: 10.1107/S2052520624005948/aw509010sup10.hkl

Structure factors: contains datablock(s) 11. DOI: 10.1107/S2052520624005948/aw509011sup11.hkl

Structure factors: contains datablock(s) 12. DOI: 10.1107/S2052520624005948/aw509012sup12.hkl

Figs S1 to S15, Tables S1 and S2. DOI: 10.1107/S2052520624005948/aw5090sup13.pdf

CCDC references: 2363717, 2363718, 2363719, 2363720, 2363721, 2363722, 2363723, 2363724, 2363725, 2363726, 2363727

## Figures and Tables

**Table:** Experiments were carried out at 100 K using a Rigaku Oxford Diffraction SuperNova diffractometer with an Atlas CCD detector. Absorption was corrected for by multi-scan methods (*CrysAlis PRO*; Rigaku Oxford Diffraction, 2015 ▸). H atoms were treated by a mixture of independent and constrained refinement.

	**1**	**2**	**3**	**4**
Crystal data				
Chemical formula	C_36_H_32_Cl_2_CuN_8_O_8_·3.3(H_2_O)	C_36_H_32_Cl_2_CuN_8_O_8_·2(CH_4_O)	C_36_H_32_Cl_2_CuN_8_O_8_·2(C_2_H_6_O)	C_36_H_32_Cl_2_CuN_8_O_8_·2(C_3_H_8_O)
*M*_r_	898.59	903.22	931.27	959.32
Crystal system, space group	Triclinic, *P* 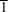	Triclinic, *P* 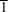	Triclinic, *P* 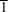	Triclinic, *P* 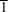
*a*, *b*, *c* (Å)	8.8398 (6), 9.9196 (8), 12.8239 (10)	9.3557 (7), 9.838 (1), 13.0238 (10)	9.3377 (3), 9.7888 (4), 12.9870 (6)	9.1329 (6), 10.0787 (7), 13.0825 (8)
α, β, γ (°)	97.633 (6), 93.948 (6), 107.385 (7)	75.710 (8), 89.649 (6), 69.657 (8)	103.114 (5), 90.344 (5), 109.408 (4)	103.496 (6), 94.562 (5), 105.229 (6)
*V* (Å^3^)	1056.59 (14)	1084.99 (17)	1086.13 (8)	1117.04 (13)
*Z*	1	1	1	1
Radiation type	Mo *K*α	Mo *K*α	Mo *K*α	Mo *K*α
μ (mm^−1^)	0.71	0.69	0.69	0.67
Crystal size (mm)	0.25 × 0.16 × 0.15	0.20 × 0.17 × 0.13	0.17 × 0.12 × 0.10	0.21 × 0.15 × 0.11

Data collection				
*T*_min_, *T*_max_	0.838, 1.000	0.896, 1.000	0.890, 1.000	0.803, 1.000
No. of measured, independent and observed [*I* > 2σ(*I*)] reflections	7899, 3692, 3323	15902, 3791, 3516	12262, 3812, 3397	10056, 4829, 4106
*R*_int_	0.026	0.035	0.027	0.025
(sin θ/λ)_max_ (Å^−1^)	0.595	0.595	0.595	0.639

Refinement				
*R*[*F*^2^ > 2σ(*F*^2^)], *wR*(*F*^2^), *S*	0.057, 0.167, 1.11	0.065, 0.172, 1.05	0.066, 0.176, 1.07	0.036, 0.094, 1.07
No. of reflections	3692	3791	3812	4829
No. of parameters	283	334	365	296
No. of restraints	19	82	423	3
Δρ_max_, Δρ_min_ (e Å^−3^)	1.52, −0.48	1.39, −0.96	1.12, −1.33	0.38, −0.46

**Table:** 

	**6**	**7**	**8**	**9**
Crystal data				
Chemical formula	C_36_H_32_Cl_2_CuN_8_O_8_·2(C_4_H_10_O)	C_36_H_32_Cl_2_CuN_8_O_8_·2(C_3_H_7_NO)	C_36_H_32_Cl_2_CuN_8_O_8_·2(C_3_H_6_O)	C_36_H_32_Cl_2_CuN_8_O_8_·2(C_4_H_8_O)
*M*_r_	987.37	985.33	955.29	983.34
Crystal system, space group	Triclinic, *P* 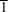	Triclinic, *P* 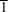	Triclinic, *P* 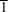	Monoclinic, *P*2_1_/*n*
*a*, *b*, *c* (Å)	9.4157 (5), 9.9855 (6), 13.6726 (7)	9.1544 (4), 9.8343 (5), 14.2557 (8)	9.4814 (4), 9.7136 (5), 13.3146 (9)	9.0943 (5), 25.0287 (17), 10.0253 (5)
α, β, γ (°)	106.181 (5), 96.455 (4), 106.527 (5)	91.493 (4), 107.948 (5), 109.075 (5)	69.117 (6), 80.660 (5), 68.833 (5)	90, 107.509 (6), 90
*V* (Å^3^)	1157.64 (12)	1142.38 (11)	1067.61 (11)	2176.2 (2)
*Z*	1	1	1	2
Radiation type	Cu *K*α	Mo *K*α	Mo *K*α	Mo *K*α
μ (mm^−1^)	2.28	0.66	0.71	0.69
Crystal size (mm)	0.16 × 0.09 × 0.09	0.25 × 0.15 × 0.15	0.16 × 0.11 × 0.10	0.23 × 0.13 × 0.13

Data collection				
*T*_min_, *T*_max_	0.815, 1.000	0.757, 1.000	0.586, 1.000	0.503, 1.000
No. of measured, independent and observed [*I* > 2σ(*I*)] reflections	7216, 4229, 3607	8820, 4905, 4337	8762, 4579, 3483	8871, 3808, 2800
*R*_int_	0.035	0.022	0.045	0.089
(sin θ/λ)_max_ (Å^−1^)	0.605	0.639	0.639	0.595

Refinement				
*R*[*F*^2^ > 2σ(*F*^2^)], *wR*(*F*^2^), *S*	0.059, 0.156, 1.06	0.037, 0.095, 1.06	0.052, 0.135, 1.06	0.062, 0.157, 1.18
No. of reflections	4229	4905	4579	3808
No. of parameters	306	303	294	301
No. of restraints	3	2	1	3
Δρ_max_, Δρ_min_ (e Å^−3^)	1.55, −0.51	0.61, −0.46	0.86, −0.86	0.87, −0.59

**Table:** 

	**10**	**11**	**12**
Crystal data			
Chemical formula	C_36_H_32_Cl_2_CuN_8_O_8_·2(C_4_H_8_O_2_)	C_36_H_32_Cl_2_CuN_8_O_8_·2(C_4_H_8_O_2_)	C_36_H_32_Cl_2_CuN_8_O_8_·C_4_H_10_O
*M*_r_	1015.34	1015.34	913.25
Crystal system, space group	Triclinic, *P* 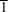	Triclinic, *P* 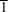	Triclinic, *P* 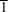
*a*, *b*, *c* (Å)	9.3679 (9), 10.2347 (8), 12.5311 (15)	10.0363 (5), 10.1867 (6), 11.9902 (6)	8.6577 (4), 9.8396 (4), 12.8200 (6)
α, β, γ (°)	79.661 (8), 84.578 (9), 73.249 (8)	83.078 (5), 82.032 (4), 70.015 (5)	97.084 (4), 93.897 (4), 108.096 (4)
*V* (Å^3^)	1130.6 (2)	1137.37 (11)	1023.61 (8)
*Z*	1	1	1
Radiation type	Mo *K*α	Mo *K*α	Mo *K*α
μ (mm^−1^)	0.67	0.67	0.73
Crystal size (mm)	0.22 × 0.10 × 0.10	0.16 × 0.13 × 0.10	0.18 × 0.11 × 0.09

Data collection			
*T*_min_, *T*_max_	0.489, 1.000	0.703, 1.000	0.812, 1.000
No. of measured, independent and observed [*I* > 2σ(*I*)] reflections	10186, 4867, 3144	9285, 4877, 4265	7915, 4386, 4098
*R*_int_	0.083	0.033	0.023
(sin θ/λ)_max_ (Å^−1^)	0.639	0.639	0.639

Refinement			
*R*[*F*^2^ > 2σ(*F*^2^)], *wR*(*F*^2^), *S*	0.069, 0.184, 1.06	0.042, 0.113, 1.07	0.031, 0.083, 1.05
No. of reflections	4867	4877	4386
No. of parameters	310	312	330
No. of restraints	2	2	10
Δρ_max_, Δρ_min_ (e Å^−3^)	0.99, −0.59	0.66, −0.51	0.37, −0.46

† Data for **5**-2(2-PrOH) are not listed.

**Table 2:** The dihedral angles (°) between the least-squares calculated planes through the atoms of the imidazole (I) and phenyl rings (R) *A* and *B* of the [Cu(ClO_4_)_2_(*L*H)_4_] molecules for the 11 solvatomorphs studied†‡§

Compound	I*A*/I*B*	I*A*/R*A*	I*B*/R*B*
**1**	83.0 (2)	17.4 (2)	27.5 (2)
**2**	81.3 (2)	13.9 (2)	27.3 (6)
**3**	82.4 (2)	12.8 (2)	31.9 (5)
**4**	89.6 (1)	20.1 (1)	13.2 (1)
**6**	84.3 (1)	18.0 (2)	11.4 (2)
**7**	72.5 (1)	2.7 (1)	22.3 (1)
**8**	73.7 (1)	18.4 (2)	35.1 (1)
**9**	87.3 (2)	11.9 (2)	31.2 (2)
**10**	85.1 (2)	11.5 (2)	21.6 (3)
**11**	88.6 (1)	10.5 (1)	6.5 (2)
**12**	80.3 (1)	16.3 (1)	22.2 (2)

† IA: Imidazole ring N1*A*/C2*A*/N3*A*/C4*A*/C5*A*; IB: Imidazole ring N1*B*/C2*B*/N3*B*/C4*B*/C5*B*; R*A*: Phenyl ring C6*A*/C7*A*/C8*A*/C9*A*/C10*A*/C11*A*; R*B*: Phenyl ring C6*B*/C7*B*/C8*B*/C9*B*/C10*B*/C11*B*.

‡ Data for compound **5**·2(2-PrOH) are not listed.

§ The numbering scheme of the complexes does not include the solvent molecules.

**Table 3:** Geometry (Å, °) of the strong hydrogen-bonding motifs in compounds **1**–**4** and **6**–**12**†

*D*—H⋯*A*	*D*—H	H⋯*A*	*D*⋯*A*	*D*—H⋯*A*
**1**				
N1*A*—H11*A*⋯O4^i^	0.84 (2)	2.06 (4)	2.836 (4)	154 (5)
N1*B*—H1*B*⋯O2^ii^	0.83 (2)	2.22 (3)	2.993 (5)	156 (4)
O5—H5*A*⋯O6*A*	0.89 (4)	2.04 (3)	2.721 (17)	133 (5)
O5—H5*A*⋯O6*B*	0.89 (4)	2.04 (4)	2.925 (15)	176 (6)
**2**				
N1*A*—H11*A*⋯O4*B*^iii^	0.86 (3)	1.98 (4)	2.763 (9)	152 (5)
N1*B*—H1*B*⋯O5^iii^	0.86 (4)	1.95 (4)	2.796 (8)	168 (5)
O5—H5⋯O2*A*^iv^	0.89 (11)	1.87 (11)	2.727 (19)	163 (9)
**3**				
N1*A*—H11*A*⋯O4*B*^i^	0.86 (2)	2.09 (4)	2.777 (8)	136 (5)
N1*B*—H1*B*⋯O5^i^	0.86 (2)	1.94 (2)	2.801 (6)	176 (6)
O5–H5*A*⋯O2*A*^v^	0.84 (2)	1.91 (2)	2.719 (13)	162 (8)
**4**				
N1*A*—H11*A*⋯O4^vi^	0.86 (2)	1.97 (2)	2.789 (3)	160 (2)
N1*B*—H1*B*⋯O5^vii^	0.86 (2)	1.95 (2)	2.804 (3)	175 (2)
O5—H5⋯O2^viii^	0.84 (3)	2.00 (3)	2.839 (3)	173 (3)
**6**				
N1*A*—H11*A*⋯O4^iii^	0.85 (4)	1.96 (4)	2.787 (5)	164 (5)
N1*B*—H1*B*⋯O5^ix^	0.86 (3)	1.96 (3)	2.808 (4)	172 (3)
O5—H5⋯O2^x^	0.83 (5)	2.03 (6)	2.854 (5)	170 (5)
**7**				
N1*A*—H11*A*⋯O4^i^	0.85 (2)	2.00 (2)	2.821 (3)	162 (2)
N1*B*—H1*B*⋯O5	0.85 (3)	1.95 (3)	2.776 (3)	164 (3)
**8**				
N1*A*—H11*A*⋯O4^x^	0.85 (2)	2.20 (3)	2.944 (4)	146 (3)
N1*B*—H1*B*⋯O5	0.87 (4)	2.01 (4)	2.854 (4)	164 (3)
**9**				
N1*A*—H11*A*⋯O4^xi^	0.85 (2)	2.05 (3)	2.859 (5)	160 (5)
N1*B*—H1*B*⋯O5^xii^	0.86 (3)	2.06 (4)	2.869 (6)	157 (4)
**10**				
N1*A*—H11*A*⋯O4^i^	0.85 (2)	1.98 (3)	2.810 (5)	163 (4)
N1*B*—H1*B*⋯O5	0.86 (4)	2.01 (4)	2.848 (6)	164 (5)
**11**				
N1*A*—H11*A*⋯O4^xiii^	0.85 (2)	2.35 (2)	2.946 (3)	128 (2)
N1*B*—H1*B*⋯O5^xiv^	0.86 (3)	1.98 (3)	2.827 (3)	170 (3)
**12**				
N1*A*—H11*A*⋯O4^iii^	0.85 (2)	2.01 (2)	2.804 (2)	154 (2)
N1*B*—H1*B*⋯O2^xv^	0.85 (2)	2.24 (2)	2.968 (2)	144 (2)

Symmetry codes: (i) *x*, *y* + 1, *z*; (ii) *x* − 1, *y*, z; (iii) *x*, *y* − 1, *z*; (iv) −*x* + 1, −*y* + 1, −*z*; (v) *x*, *y* − 1, z; (vi) −*x*, −*y* − 1, −*z*; (vii) −*x*, −*y*, − *z*; (viii) − *x* + 1, − *y*, − *z*; (ix) *x* − 1, *y* − 1, *y* − 1; (x) − *x*, −*y* + 1, −*z* + 1; (xi) *x*, *y*, *y* − 1; (xii) *x* + 1, *y*, *z*; (xiii) −*x* + 1, − *y* + 1, −*z* + 2; (xiv) −*x* + 2, −*y* + 1, −*z* + 2; (xv) −*x*, − *y* + 1, −*z*.

† The numbering scheme of the complexes does not include the solvent molecules.
