# Supplementary material for: Solvatomorphism in a series of copper(II) complexes with the 5-phenyl­imidazole/perchlorate system as ligands
Source: Acta Crystallogr B Struct Sci Cryst Eng Mater. 2024 Jul 30;80(Pt 4):347–59. doi: 10.1107/S2052520624005948 (PMC11301897; doi:10.1107/S2052520624005948)
Supplement: Supplementary file 13 [file b-80-00347-sup13.pdf]

STRUCTURAL SCIENCE
CRYSTAL ENGINEERING
MATERIALS

Volume 80 (2024)

Supporting information for article:

Solvatomorphism in a series of copper(II) complexes with the 5-phenylimidazole/perchlorate system as ligands

Edward Loukopoulos, Constantina Papatriantafyllopoulou, Eleni Moushi, Alexandros A. Kitos, Anastasios J. Tasiopoulos, Spyros P. Perlepes and Vassilios Nastopoulos

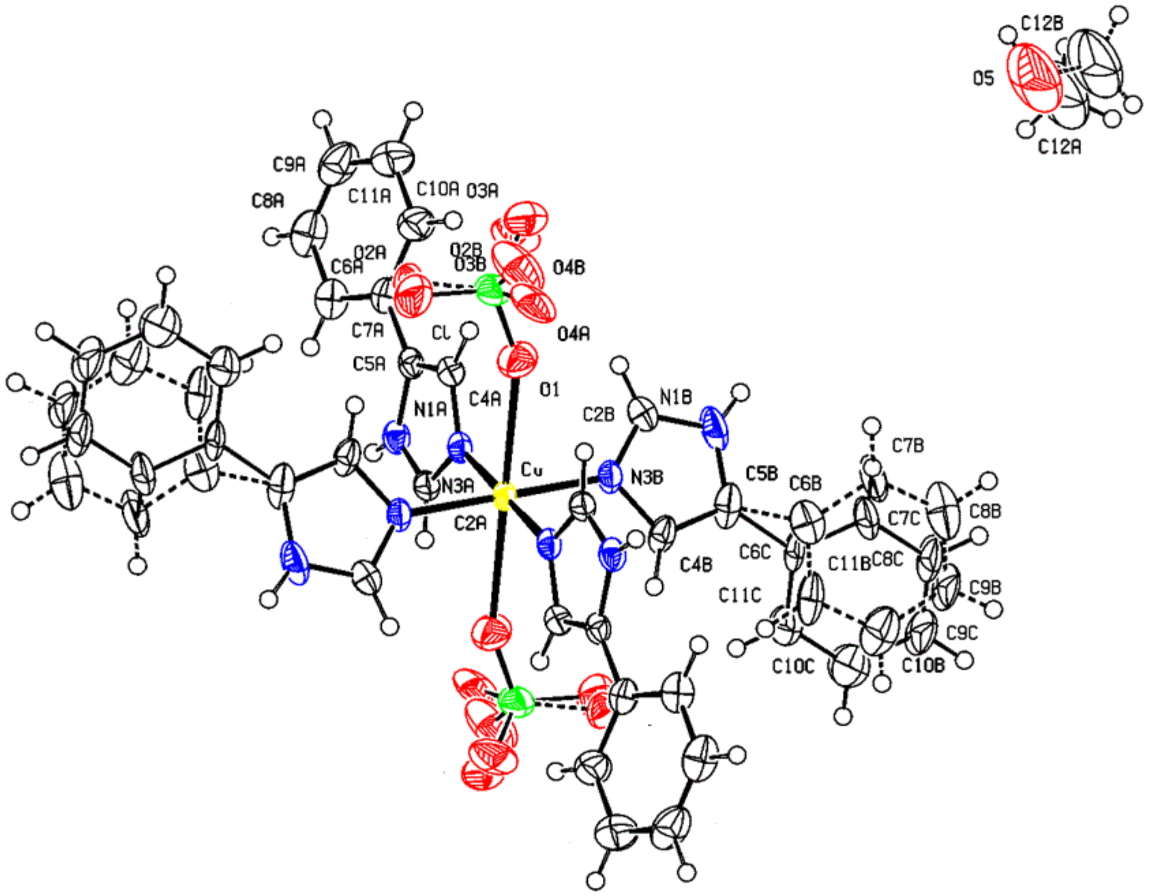

Figure S1 A view of the structure of compound **2**·2MeOH with atom labelling and displacement ellipsoids drawn at the 50% probability level. Atoms generated by symmetry through the inversion centre located on the copper ion are not labelled. Both orientations of the disordered phenyl rings, the ClO_4^- ion and the MeOH solvent molecule are shown.

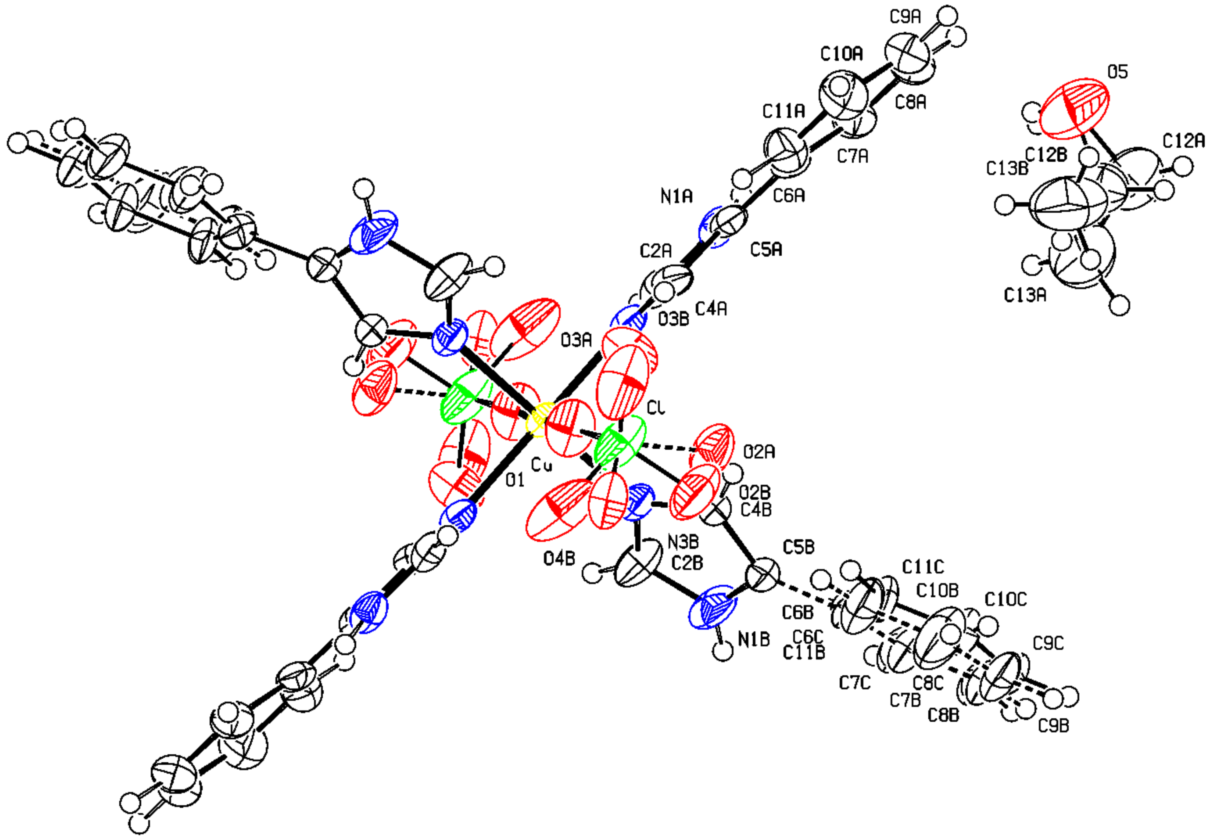

Figure S2 A view of the structure of compound **3**·2EtOH with atom labelling and displacement ellipsoids drawn at the 50% probability level. Symmetry-related atoms through the inversion centre located on the copper ion site are not labelled. Both orientations of the disordered phenyl rings, the ClO_4^- ion and the EtOH solvent molecule are shown.

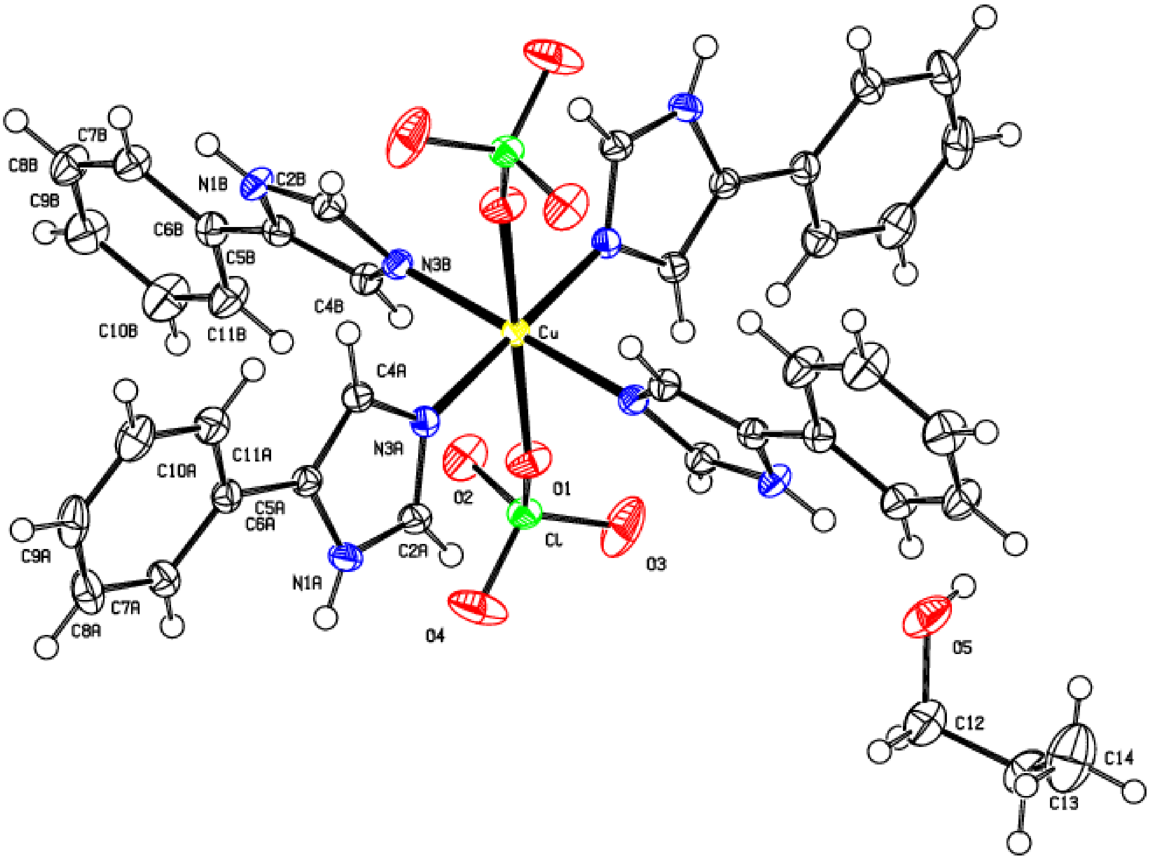

Figure S3 A view of the structure of compound **4**·2(1-PrOH) with atom labelling and displacement ellipsoids drawn at the 50% probability level. Symmetry-related atoms through the inversion centre located on the copper ion site are not labelled.

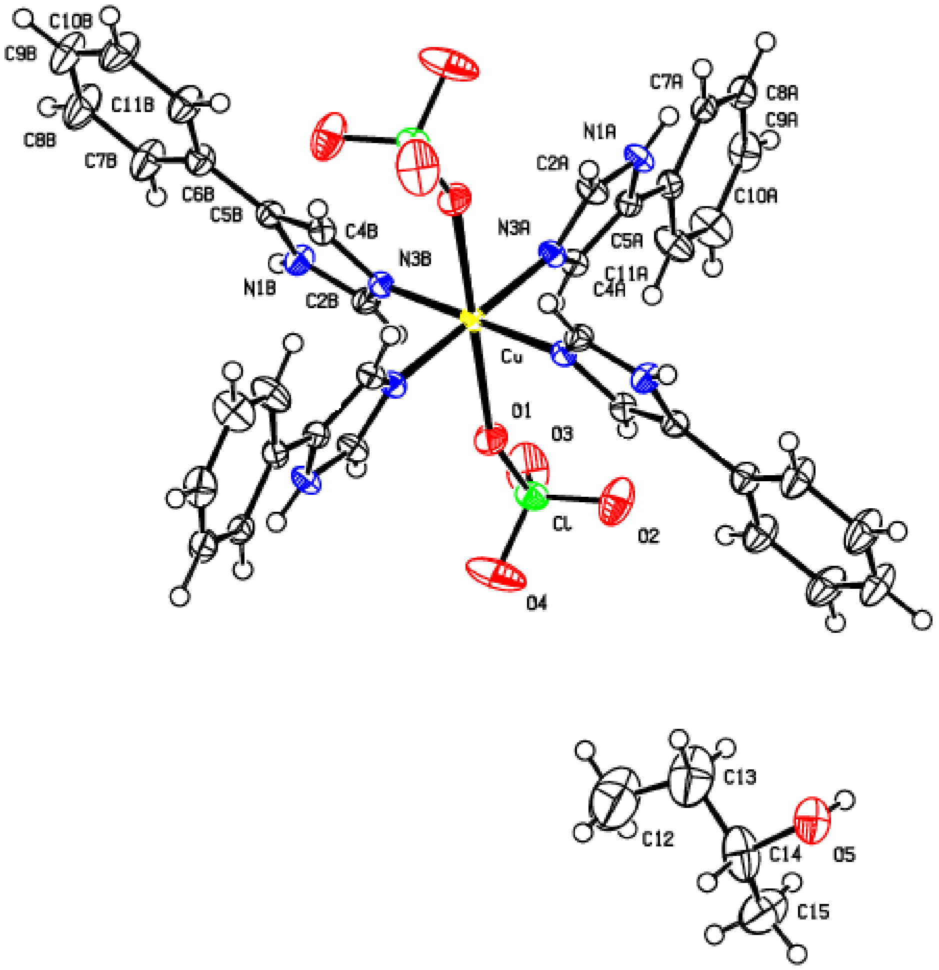

Figure S4 A view of the structure of compound **6**·2(2-BuOH) with atom labelling and displacement ellipsoids drawn at the 50% probability level. Symmetry-related atoms through the inversion centre located on the copper atom site are not labelled.

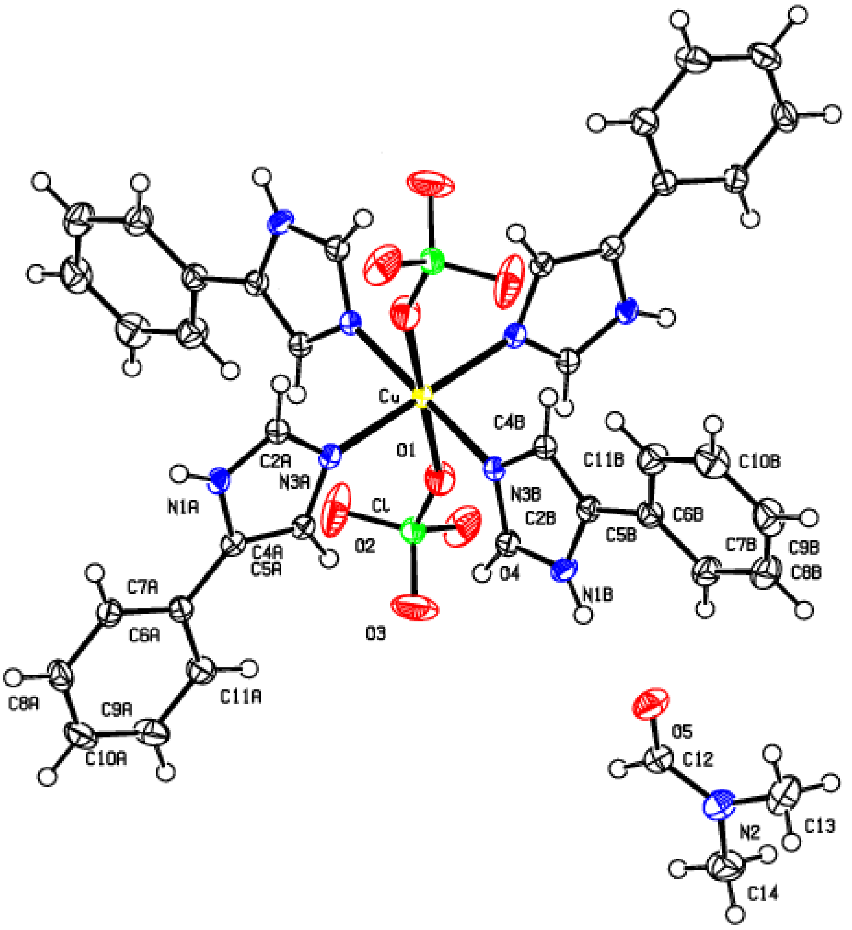

Figure S5 A view of the structure of compound **7**·2DMF with atom labelling and displacement ellipsoids drawn at the 50% probability level. Symmetry-related atoms through the inversion centre located on the copper ion site are not labelled.

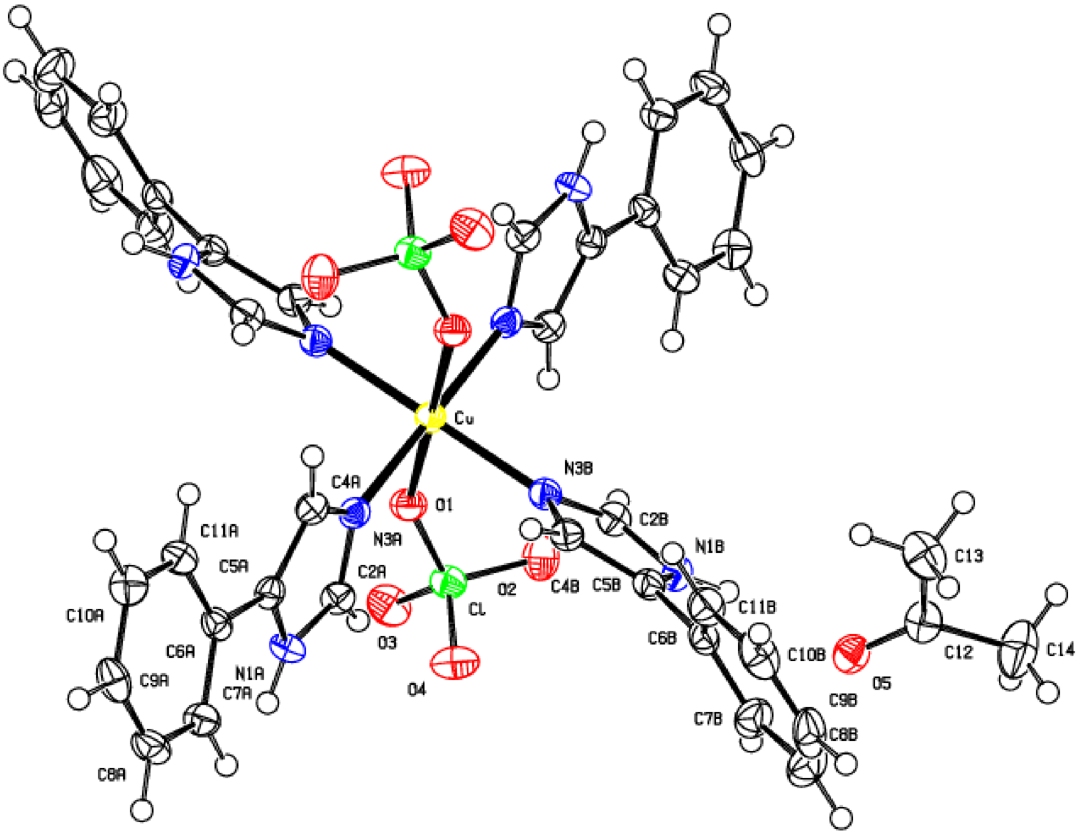

Figure S6 A view of the structure of compound **8**·2Me$_2$CO with atom labelling and displacement ellipsoids drawn at the 50% probability level. Symmetry-related atoms through the inversion centre located on the copper ion site are not labelled.

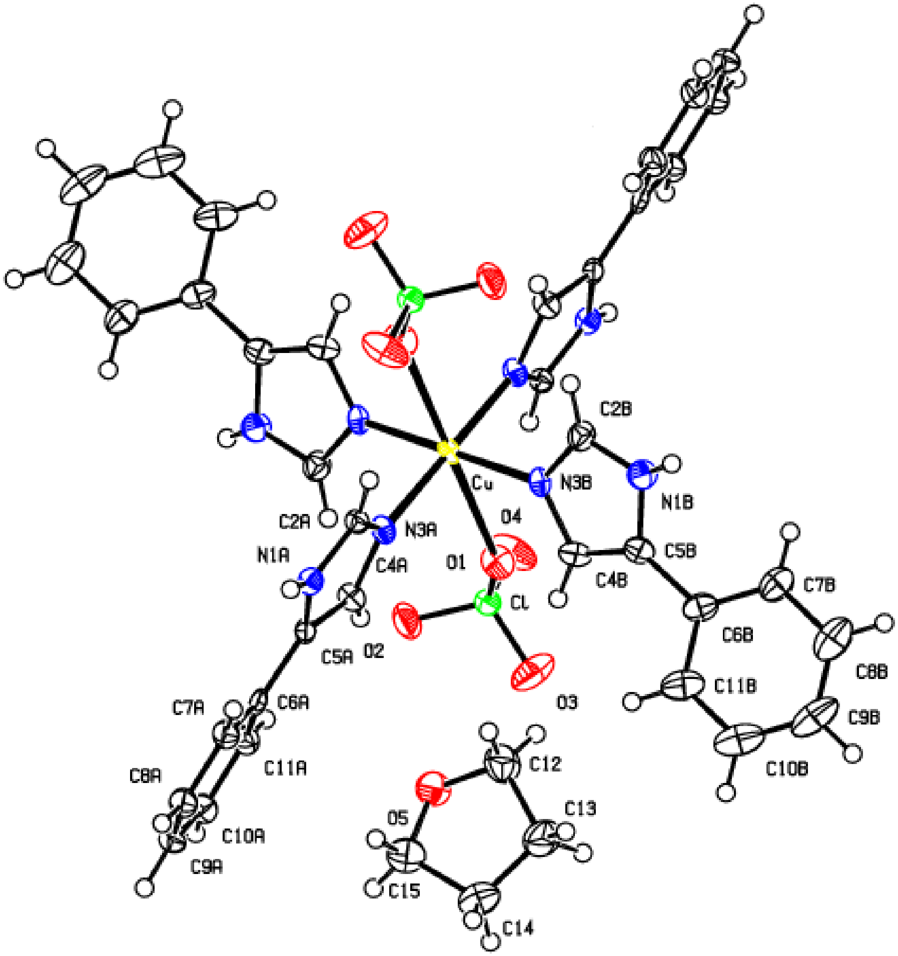

Figure S7 A view of the structure of compound **9**·2THF with atom labelling and displacement ellipsoids drawn at the 50% probability level. Symmetry-related atoms through the inversion centre located on the copper ion site are not labelled.

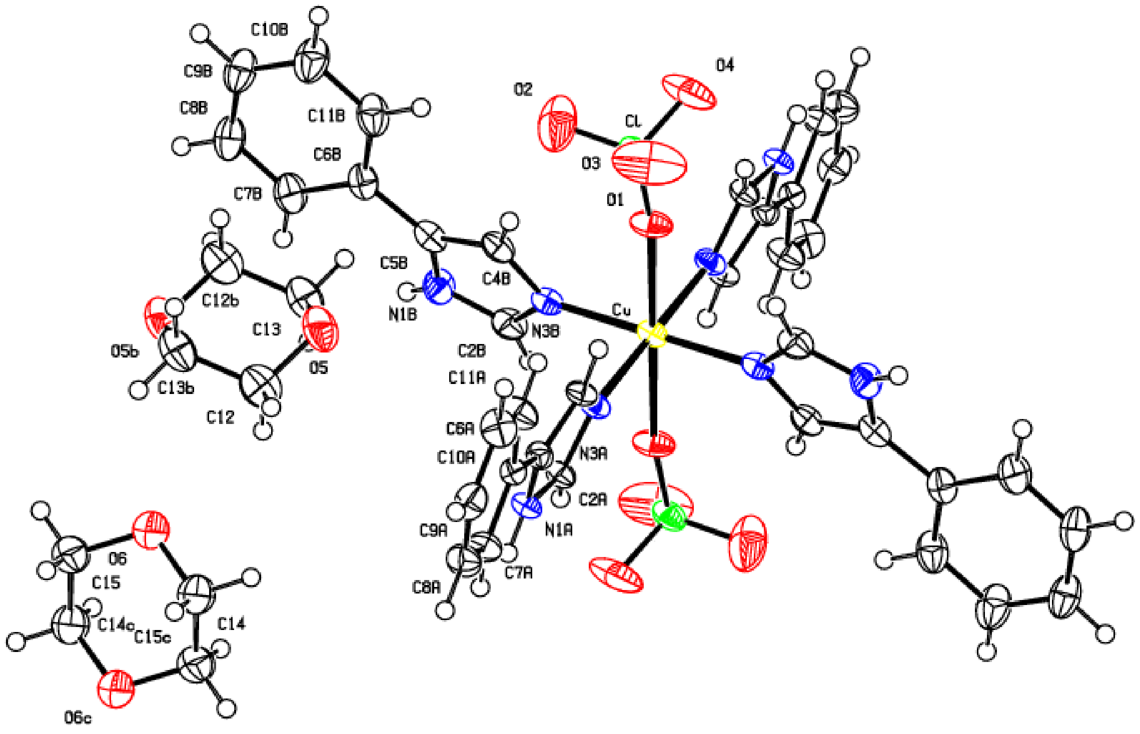

Figure S8 A view of the structure of compound **10**·2(1,4-dioxane) with atom labelling and displacement ellipsoids drawn at the 50% probability level. Symmetry-related atoms through the inversion centre located on the copper ion site are not labelled.

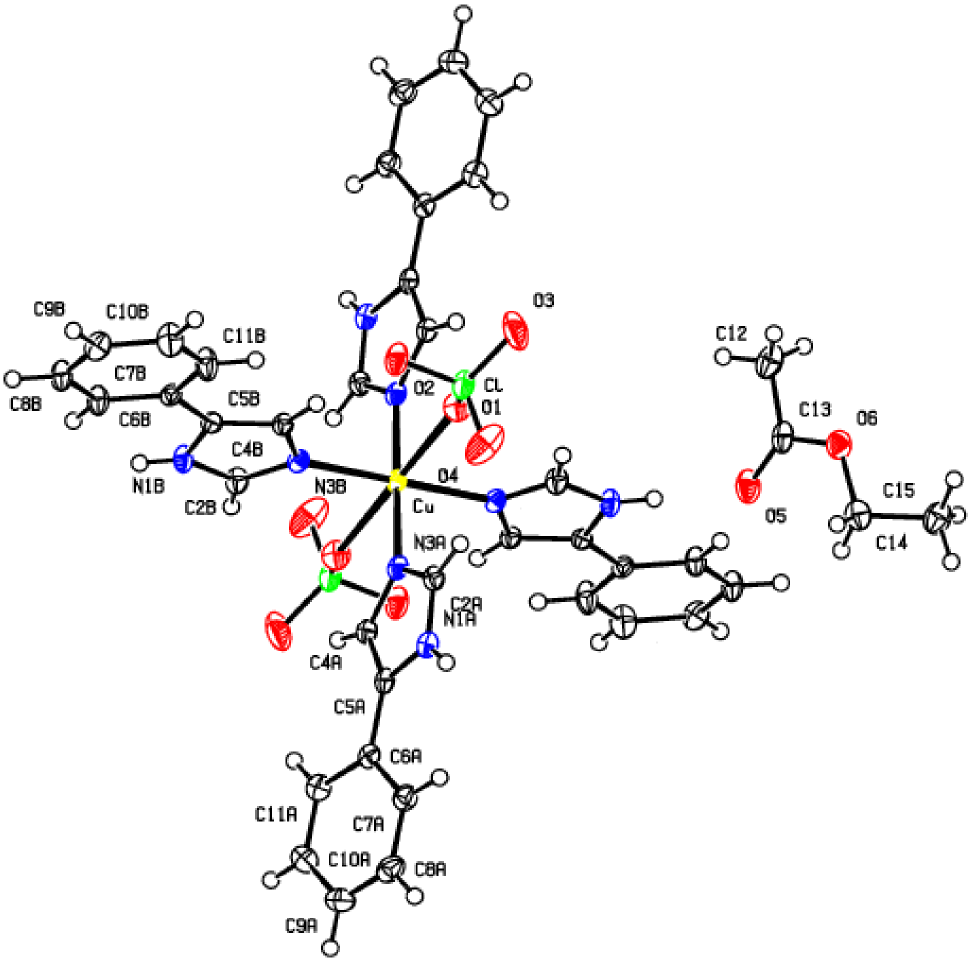

Figure S9 A view of the structure of compound **11**·2EtOAc with atom labelling and displacement ellipsoids drawn at the 50% probability level. Symmetry-related atoms through the inversion centre located on the copper ion site are not labelled.

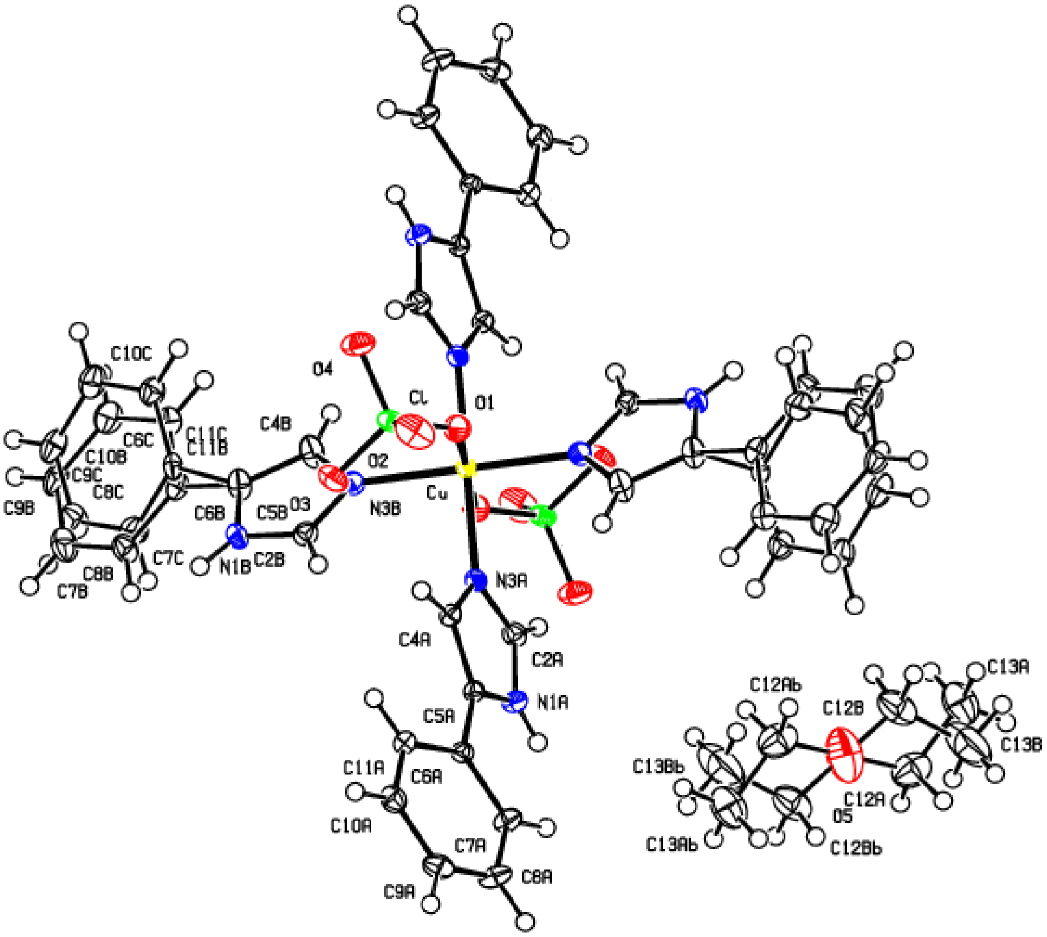

Figure S10 A view of the structure of compound **12**·Et_2O with atom labelling and displacement ellipsoids drawn at the 50% probability level. Symmetry-related atoms through the inversion centre located on the copper ion site are not labelled. Both orientations of the disordered phenyl rings and the Et_2O solvent molecule are shown.

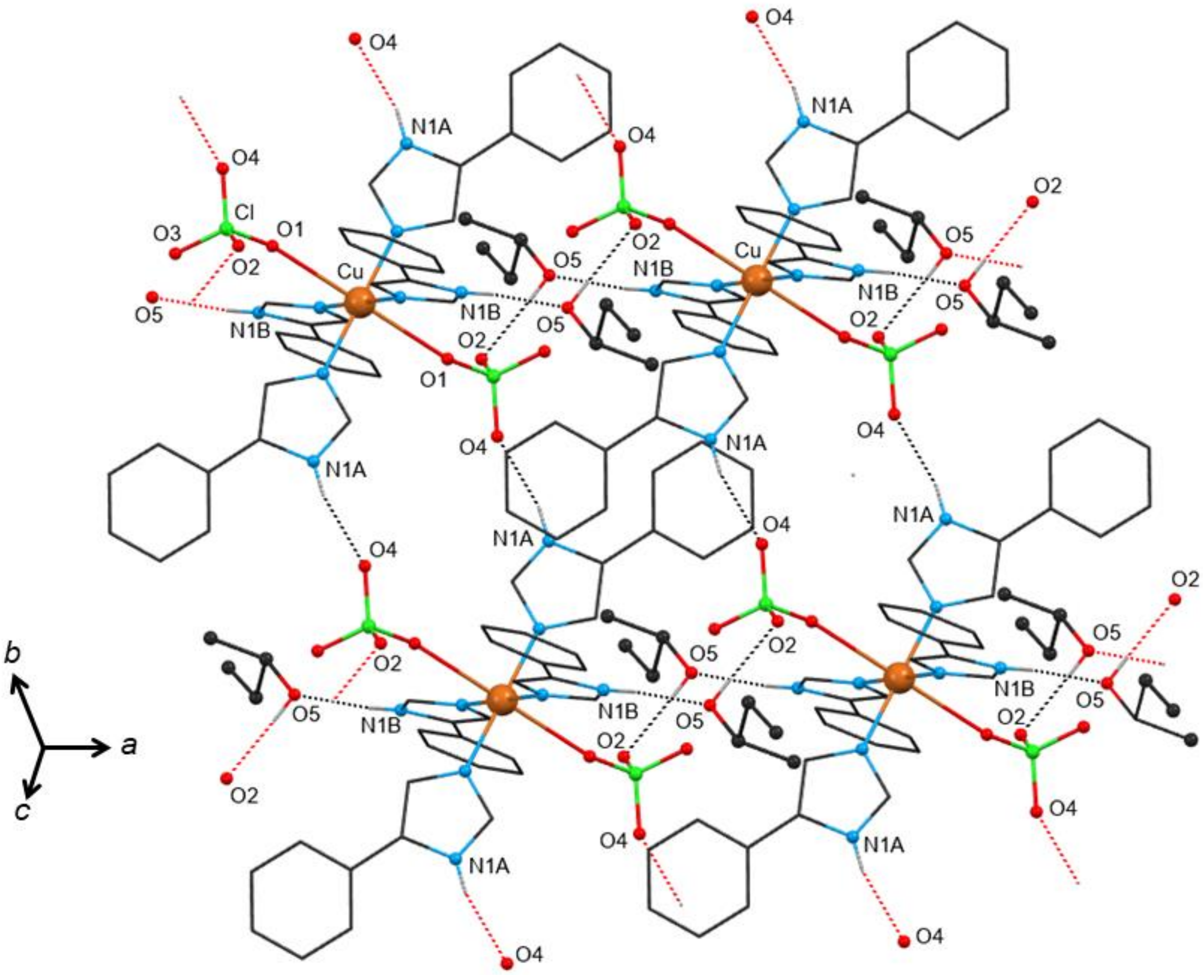

Figure S11 View of a 2D layer of the crystal structure of solvatomorph **6**·2(2-BuOH) organized by hydrogen-bonding motifs (see Table 3). The atoms of the 2-BuOH solvent are drawn in ball-and-stick style (oxygen atom: O5). Only the H-atoms involved in strong intermolecular interactions are shown.

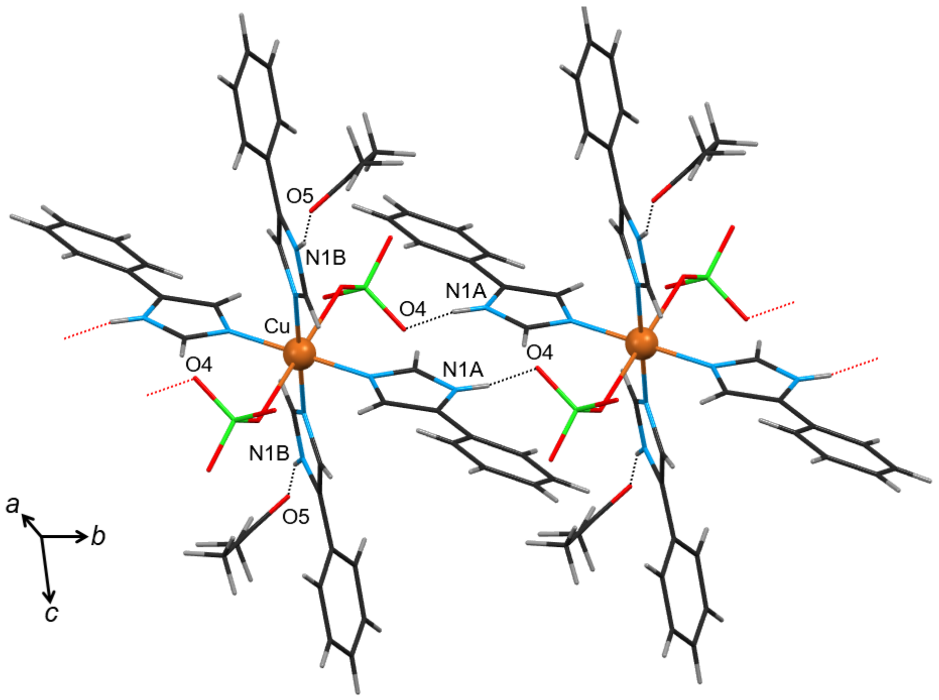

(a)

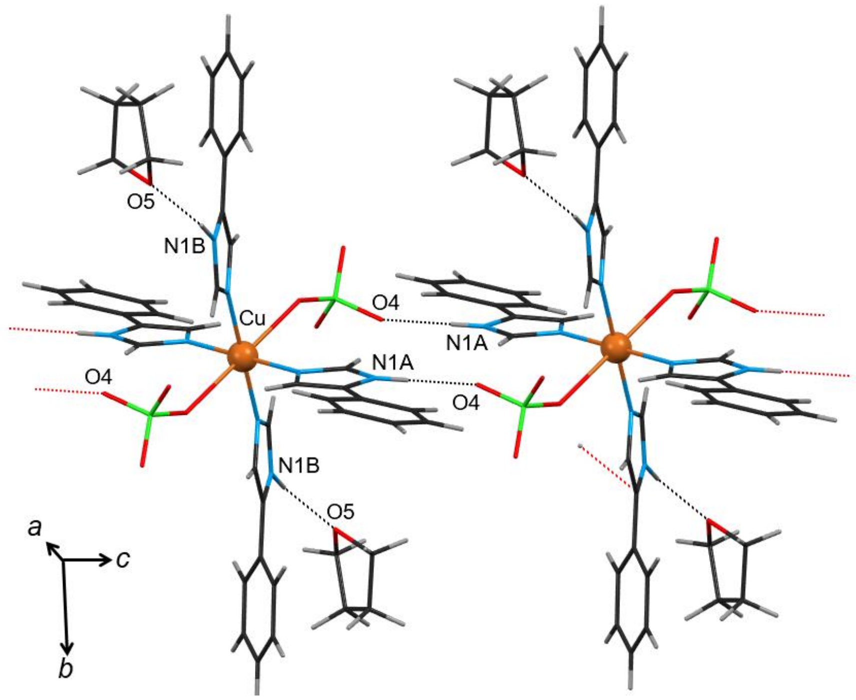

(b)

Figure S12 1D tapes in the crystal structure of (a) solvatomorph **8**·$2Me_2CO$ and (b) **9**·2THF formed by the $[Cu(ClO_4)_2(LH)_4]$ molecules *via* N1A−H1A···$O4_{perchlorate}$ synthons (see Table 3). The Me_2CO and THF solvents (bearing the O5 atoms) are only terminally hydrogen-bonded to the host complexes.

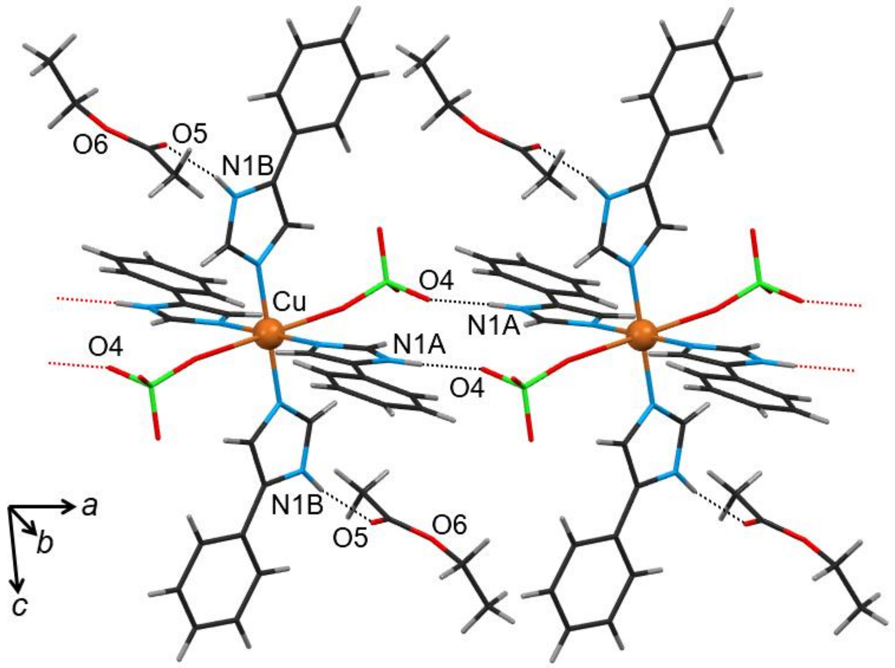

Figure S13 1D tapes in the crystal structure of solvatomorph **11**·2EtOAc formed by the [$Cu(ClO_4)_2(LH)_4$] molecules *via* N1A−H1A···O4$_{perchlorate}$ synthons (see Table 3). The EtOAc solvent (bearing O5 and O6 atoms) is only terminally hydrogen-bonded to the host complexes.

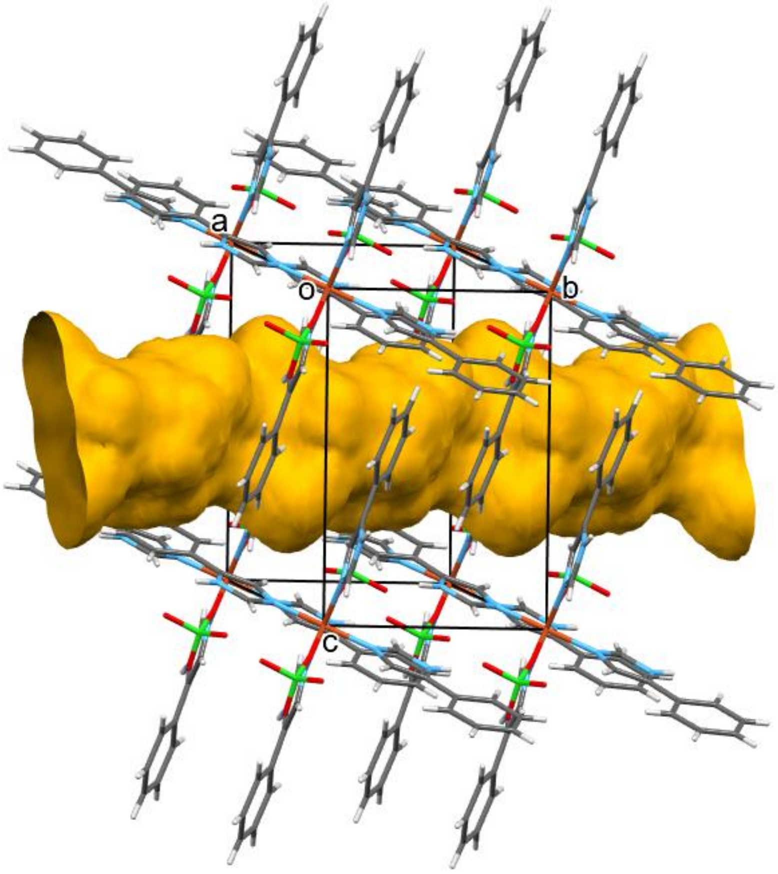

Figure S14 View of the 3D structure of solvatomorph **10**·2(1,4-dioxane). The dioxane solvent molecules have been removed and the channels containing them along the *b*-axis of the unit cell are drawn in yellow.

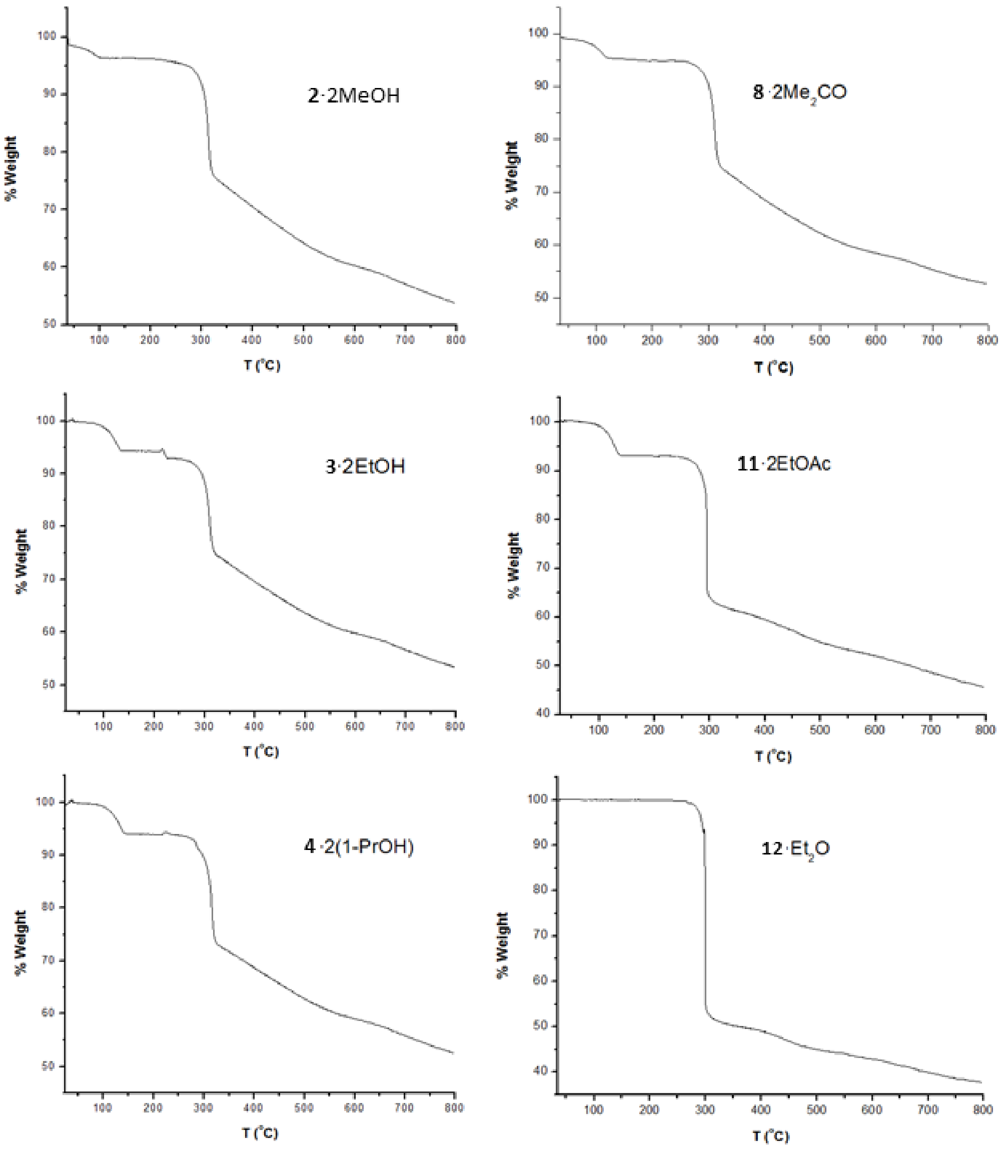

Figure S15 TGA plots for selected complexes of this work.

Table S1 π···π stacking distances (Å) and angles (º) in complexes **1**–**4** and **6**–**12**.

Structure	Rings involved [a]	Distance between ring centroids	Perpendicular distance between ring planes	Centroid offset	Dihedral angle between ring mean-planes
**1**	B2–B2^{i}	3.883(3)	3.505(2)	1.671	0.0
	A1–A2^{ii}	3.862(2)	3.158(2)	1.190	17.5(2)
**2**	A1–A2^{iii}	3.745(2)	3.186(2)	1.194	13.8(2)
	B2–C2^{iv}	3.821(2)	3.620(6)	0.963	6.3(6)
**3**	C2–C2^{v}	3.578(5)	3.392(4)	1.137	0.0
	A1–B2^{v}	3.788(6)	3.635(4)	0.877	7.1(5)
**4**	B2–B2^{vi}	4.059(2)	3.636(1)	1.805	0.0
**6**	A1–A2^{vii}	3.938(2)	2.946(2)	1.599	18.0(2)
	B2–B2^{vi}	3.993(2)	3.595(2)	1.739	0.0
**7**	A1–A2^{viii}	3.666(2)	3.331(8)	1.594	2.7(1)
	B2–B2^{ix}	4.033(2)	3.634(1)	1.750	0.0
**8**	A2–A1^{x}	3.760(2)	3.244(2)	0.856	18.4(2)
	A1–B2^{xi}	3.889(2)	3.500(2)	1.694	0.02(2)
**9**	A2–A1^{ix}	3.685(3)	3.090(2)	1.329	11.9(3)
**10**	A1–A2^{xii}	3.866(2)	3.032(2)	1.749	11.5(2)
**11**	A2–A1^{ix}	3.597(2)	3.191(1)	1.064	10.4(2)
	B2–B1^{xiii}	3.881(2)	3.478(2)	1.534	6.5(2)
**12**	A2–A1^{v}	3.953(2)	3.266(2)	1.318	16.3(1)

[a] Ring A1: N1A to C5A; Ring A2: C6A to C11A; Ring B1: N1B to C5B; Ring B2: C6B to C11B; Ring C2: C6C to C11C.

Symmetry codes: (i) –x–1, –y, –z–1; (ii) –x+1, –y+1, –z; (iii) –x+1, –y–1, –z; (iv) –x, –y, –z+1; (v) –x, –y, –z; (vi) –x–1, –y–1, –z–1; (vii) –x –1, –y–1, –z; (viii) –x+2, –y+2, –z+1; (ix) –x+1, –y+1, –z+1; (x) –x –1, –y+1, –z+1; (xi) –x, –y+1, –z; (xii) –x–1, –y+1, –z; (xiii) –x+2, –y+1, –z+3.

Table S2 C–H···π interaction parameters (Å, º) in compounds **1–4** and **6–12**.

Structure	D–H···A[a]	H···Cg[b]	H···(ring plane)[c]	γ[d]	C···Cg	X–H···Cg
**1**	C8A–H8A···Cg1^{i}	2.82	2.75	13.2	3.66	148
	C8B–H8B···Cg2^{ii}	2.97	2.87	14.9	3.77	143
**2**	C8A–H8A···Cg1^{iii}	2.64	2.61	7.9	3.49	151
	C9A–H9A···Cg3^{iv}	2.83	2.63	21.5	3.68	150
**3**	C8A–H8A···Cg1^{i}	2.67	2.65	7.0	3.54	153
	C9C–H9C···Cg3^{v}	2.77	2.66	16.5	3.66	155
	C13A–H13A···Cg4^{v}	2.89	2.81	13.4	3.48	120
**4**	C8A–H8A···Cg1^{vi}	2.67	2.63	10.3	3.58	161
	C8B–H8B···Cg2^{vii}	2.73	2.70	8.3	3.57	148
**6**	C8A–H8A···Cg1^{i}	2.69	2.65	10.3	3.62	148
	C8B–H8B···Cg2^{ii}	2.89	2.75	17.9	3.69	143
**7**	C8A–H8A···Cg1^{viii}	2.67	2.65	7.7	3.60	164
	C14–H14B···Cg4^{ix}	2.97	2.86	16.1	3.83	146
**8**	C8A–H8A···Cg1^{x}	2.75	2.74	3.2	3.54	142
**9**	C8A–H8A···Cg1^{xi}	2.66	2.63	8.6	3.56	160
	C14–H14B···Cg4^{xii}	2.99	2.84	18.3	3.78	138
	C15–H15B···Cg2	2.99	2.87	15.9	3.72	132
**10**	C8A–H8A···Cg1^{x}	2.74	2.70	9.3	3.66	164
	C14–H14A···Cg4^{ix}	2.80	2.74	12.1	3.75	161
**11**	C8A–H8A···Cg1^{xiii}	2.81	2.66	18.8	3.67	151
	C8B–H8B···Cg3^{iv}	2.94	2.88	11.5	3.72	141
**12**	C8A–H8A···Cg1^{xiv}	2.85	2.77	13.7	3.65	143
	C8A–H8A···Cg2^{vii}	2.92	2.80	16.4	3.72	143

[a] Ring 1: N1B to C5B; Ring 2: C6A to C11A; Ring 3: N1A to C5A; Ring 4: C6B to C11B.

[b] Cg is the centroid (centre of gravity) of the ring.

[c] perpendicular distance of H to ring plane.

[d] γ is the angle defined by the H···Cg line and the perpendicular H···(ring plane) line.

Symmetry codes: (i) 1+x, 1+y, z; (ii) –x, –y, –z–1; (iii) –x+1, –y–1, –z; (iv) x, y, z+1; (v) –x, –y, –z; (vi) –x–1, –y–1, –z; (vii) x, y, z–1; (viii) –x+2, –y+2, –z+1; (ix) 1+x, y, z; (x) x–1, y+1, z; (xi) x–1, y, z–1; (xii) x–1, y, z; (xiii) –x+1, –y+2, –z+2; (xiv) –x, –y, –z.
